# A frontopolar-temporal circuit determines the impact of social information in macaque decision making

**DOI:** 10.1016/j.neuron.2023.09.035

**Published:** 2024-01-03

**Authors:** Ali Mahmoodi, Caroline Harbison, Alessandro Bongioanni, Andrew Emberton, Lea Roumazeilles, Jerome Sallet, Nima Khalighinejad, Matthew F.S. Rushworth

**Affiliations:** 1 Wellcome Centre for Integrative Neuroimaging, Department of Experimental Psychology, University of Oxford, Oxford, UK; 2 Cognitive Neuroimaging Unit, CEA, INSERM, Université Paris-Saclay, NeuroSpin Center, 91191 Gif/Yvette, France; 3 Department of Biomedical Services, University of Oxford, Oxford, UK; 4 Univ Lyon, Université Lyon 1, Inserm, Stem Cell and Brain Research Institute, U1208 Bron, France

**Keywords:** social information use, macaques, dorsomedial frontopolar cortex, fMRI, transcranial ultrasound stimulation

## Abstract

When choosing, primates are guided not only by personal experience of objects but also by social information such as others’ attitudes toward the objects. Crucially, both sources of information—personal and socially derived—vary in reliability. To choose optimally, one must sometimes override choice guidance by personal experience and follow social cues instead, and sometimes one must do the opposite. The dorsomedial frontopolar cortex (dmFPC) tracks reliability of social information and determines whether it will be attended to guide behavior. To do this, dmFPC activity enters specific patterns of interaction with a region in the mid-superior temporal sulcus (mSTS). Reversible disruption of dmFPC activity with transcranial ultrasound stimulation (TUS) led macaques to fail to be guided by social information when it was reliable but to be more likely to use it when it was unreliable. By contrast, mSTS disruption uniformly downregulated the impact of social information on behavior.

## Highlights

• Macaques’ dmFPC activity changes with the reliability of social information
• mSTS-dmFPC interaction occurs when social information influences behavior
• Disruption of dmFPC decreases the impact of social information when it is reliable
• Disruption of dmFPC increases the impact of social information when it is unreliable

## Teaser

Mahmoodi et al. identify a neural circuit in macaques that comprises dorsomedial frontopolar cortex (dmFPC) and mid-superior temporal sulcus (mSTS) for regulating the impact of social information according to its reliability. Disruption of dmFPC and mSTS produces distinct changes in social information use.

## Introduction

When faced with a choice, we often rely on several cues to guide our decision. In some cases, our behavior is directed by our own prior experience. For example, when choosing a meal at a restaurant, we may select a dish that we enjoyed previously. Sometimes, however, we may have reservations about the reliability or relevance of such experience. In this example, when traveling abroad, we instead place particular reliance on the opinions of others when choosing a dish. Human studies have demonstrated the impact of such social influence on behavior,^1^^,^^2^^,^^3^^,^^4^^,^^5^^,^^6^^,^^7^ whereas similar patterns have been observed in other non-human primates. When vervet monkeys in the wild are offered two different foods that are identical in taste but different in color, they choose only the food color that they observe others eating.^8^ Moreover, like the human diner abroad, vervets become more susceptible to social influence (i.e., opinion of others) when in unfamiliar territory.

Stimulus enhancement is believed to be the underlying mechanism of social influence in monkeys.^9^ During stimulus enhancement, a monkey directs its behavior more toward a stimulus or object when it sees others doing the same.^9^ Consistent with this account, neural activity in human and non-human primates reflects aspects of the actions of others,^2^^,^^10^^,^^11^^,^^12^^,^^13^ but there is uncertainty about the impact that such activity has on the observer’s behavior.^14^ In humans, social information has a higher impact when it is more reliable or when the individuals are less certain about their own decisions.^7^^,^^15^^,^^16^ Whether and how non-human primates shift flexibly from private to social information remain elusive. Here, we aim to address these questions, examining the way in which social influence may be accepted in some circumstances but not in others and its neural basis.

Another animal's face direction is an important cue in social situations.^17^^,^^18^ Making eye contact with other animals and following their face direction can be used to infer what they might be about to do.^19^^,^^20^^,^^21^^,^^22^ Monitoring where another animal’s attention is directed may also be used to gain information about the environment that can be exploited by the observer. However, just like other sources of information, the reliability of social information varies. How the non-human primate’s brain evaluates the reliability of social information to improve its decision making has not been studied. To search for brain mechanisms for evaluating and using social information, we trained macaques to perform a series of behavioral tasks in a magnetic resonance imaging (MRI) scanner while we recorded activity across the whole brain. We then used minimally invasive transcranial ultrasound stimulation (TUS) to examine the impact of transient neural disruption on behavior.

## Results

### Experiment 1

**Figure 1:**
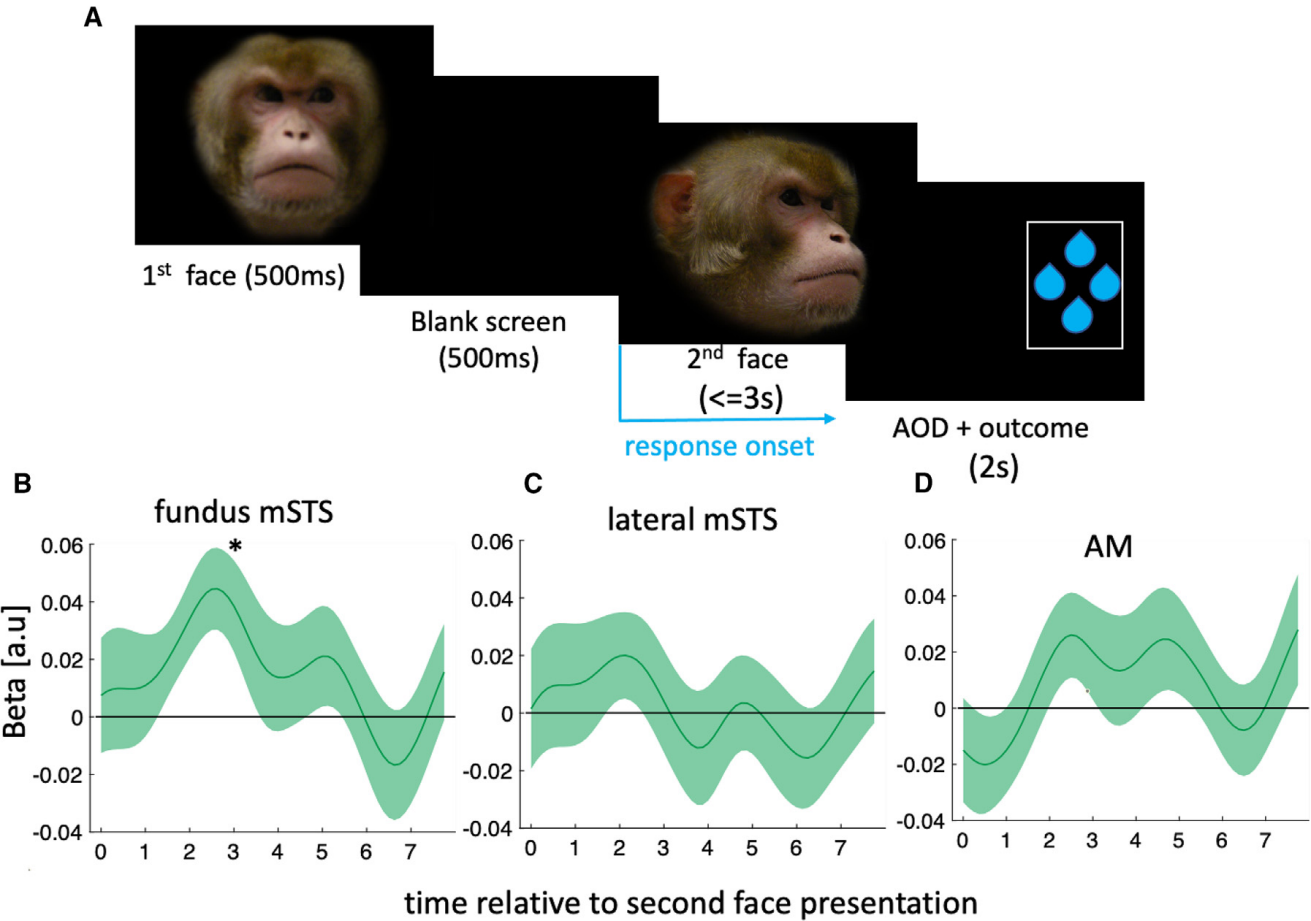
Experiment 1: face direction is encoded in mSTS (A) Two faces appeared consecutively (separated by 500 ms blank screen delay). Animals had 3 s from onset of the second face to choose which side it was facing. (B–D) Response of mSTS fundus (B), lateral mSTS (C), and AM (D) to identical versus different face direction. y axis indicates $\beta_{1}$ in Equation 7. In (B)–(D), the line and the shaded area represent mean and standard error of the mean (SEM), respectively.^∗^$p_{FDR}$ < 0.05.

Before examining how social influence is exerted in decision making, or is upregulated or downregulated, we sought to identify brain areas encoding other animals’ face direction. On each trial, a monkey’s face appeared at the screen center for 500 ms (first face), followed by 500 ms of blank screen. The face was either *neutral* (gazing forward) or *directed* (gazing left or right). Afterward, a second face, always directed, appeared. Animals chose the side the second face was gazing at (Figure 1A). The direction of the second face could have been similar or dissimilar to the first face. The experimental design was optimized to exploit repetition suppression (a reduction in neural activity due to repeated presentation of a stimulus feature) to find brain areas encoding face direction by looking for areas in which activity was reduced when the second face direction was the same as, as opposed to different to, the first face. We primarily focused on three regions of interest (ROIs) in or adjacent to the superior temporal sulcus (STS) known to contain face-responsive neurons. The first two ROIs were in the fundus of the mid-portion of the STS (mSTS, Figure S1 for coordinates) and a laterally adjacent region where STS meets the inferior temporal gyrus (coordinates taken from an independent study^23^). A third region was close to the anterior medial (AM) face patch.^24^^,^^25^

In all time-course analyses, we applied Holm-Bonferroni correction (HBC) for multiple comparison across ROIs and false discovery rate (FDR) across time points. Examination of ROI activity using repetition suppression (Figure S1 legend) indicated that at the time of the second face, only mSTS fundus activity exhibited significant face direction repetition suppression (Figure 1B, $p_{FDR}$ = 0.03, HBC), whereas neither the more lateral mSTS ($p_{FDR}$ > 0.05) nor AM ($p_{FDR}$ > 0.05) reached significance (Figures 1C and 1D). The results suggest that mSTS fundus (henceforth mSTS) encodes face direction (Figure S1 summarizes whole-brain results).

### Experiment 2

**Figure 2:**
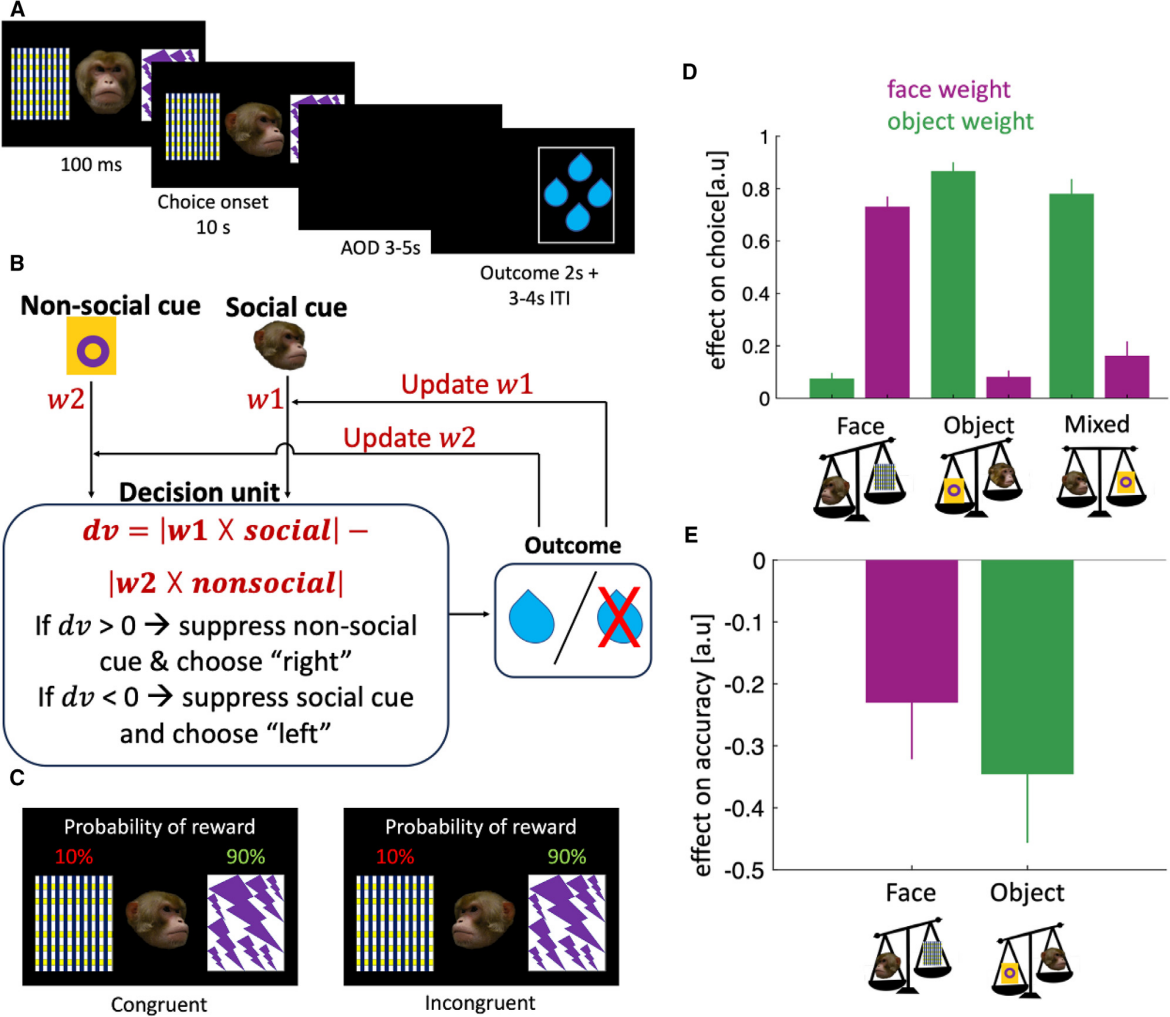
Experiment 2: task design and behavioral results (A) Animals were presented with two objects on either side of the screen and a face gazing at one of the two objects. (B) The main computational problems in social situations can be divided into learning stage and decision stage. In the learning stage, the reliability of social information should be learned (using any learning algorithm). In the decision stage, the learned reliability should be used to regulate the weight of the social information. In this study, the computational problem that the animals encountered during the task was to solve the decision stage as the animals were tested once they reached near optimal performance. Therefore, their task was to weigh each source of information according to its reliability and choose the more reliable information. (C) In all conditions, a trial was marked as incongruent if the face was directed away from the better of the two objects. In these trials, it was critical to weigh each source of information according to its reliability. (D) Beta coefficients from linear mixed-effect models are plotted for different experimental conditions ($\beta_{2}$ and $\beta_{3}$ in Equation 1). (E) Beta coefficients indicating the impact of incongruency on accuracy ($\beta_{2}$ in Equation 2). In (D) and (E) error bars indicate 95% confidence interval.

Next, we examined how animals switch between reliance and non-reliance on social information during decision making (Figures 2A and 2B). Each monkey (n = 3) performed 16 functional MRI (fMRI) sessions. At the beginning of each trial, an image of a monkey’s face appeared in the center of the screen gazing forward, with an object on either side of the face (Figure 2A). After 100 ms, the face turned to one object, and animals chose the side they thought would lead to reward. The experiment was divided into three conditions/blocks, each containing 50 trials. In the face condition, face reliability was high (the face gazed at the rewarded object in 90% of trials), whereas the better and the worse objects each had low reliabilities. In the object condition, object reliability was high (90%), whereas face reliability was low. In the mixed condition, both face and object reliabilities were high. Prior training with both face and object stimuli ensured that animals’ decisions reflected the reward probabilities of the different pairs of objects and faces (STAR Methods). We used different pairs of objects and faces for the reliable and unreliable conditions (i.e., one pair of objects for the object and mixed conditions and a different pair for the face condition, and one face for the face and mixed conditions and another for the object condition). Thus, macaques could infer the current condition by the object and face identities and their associated reliabilities. A distinct feature of our task design is that in some trials (regardless of the condition), the face was directed away from the better of the two objects. We labeled these trials as “incongruent” trials, as opposed to “congruent” trials where the face was directed toward the better of the two objects (Figure 2C). There was no conflict between the two sources of information in the congruent trials, whereas in the incongruent trials, there was conflict. The animals should have decided which source of information to follow according to the reliability of each source.

We used mixed-effects logistic regression to quantify the impact of faces and objects on choices in each condition (linear mixed-effect model [LMM1] in STAR Methods). In the face condition, choice was predominantly influenced by face direction (Figure 2D, β ± 95% confidence interval [CI] = 0.73 ± 0.04, F(1, 45) = 1,229, p < 0.001); however, interestingly, there was also a significant effect of object (Figure 2D, β ± 95% CI = 0.07 ± 0.03, F(1, 43) = 26, p < 0.001). This pattern was reversed in the object condition: choices were predominantly influenced by objects (Figure 2D, β ± 95% CI = 0.86 ± 0.03, F(1, 46) = 2,134, p < 0.001), but there was also a small but significant effect of face (β ± 95% CI = 0.08 ± 0.03, F(1, 52) = 37, p < 0.001). The choice pattern in the mixed condition resembled that seen in the object condition; choices were largely determined by objects (Figure 2D, β ± 95% CI = 0.78 ± 0.07, F(1, 46) = 569, p < 0.001), but there was also a significant effect of face (β ± 95% CI = 0.16 ± 0.06, F(1, 46) = 33, p < 0.001). The greater reliance placed on objects in the mixed condition, even when face and object reliabilities were equivalent, implies that choosing based on object information was the animals’ default strategy, and they only deviated from it when object information was less reliable than face information. The effect of face in the object condition and the effect of object in the face condition imply that accuracy should have decreased in incongruent trials (Figure 2C, trials in which the face was not gazing at the more reliable object; LMM2 in STAR Methods). Consistent with our prediction, in both conditions, incongruency impaired accuracy (Figure 2E, face condition: β ± 95% CI = −0.23 ± 0.09, F(1, 45) = 24, p < 0.001, object condition: β ± 95% CI = −0.34 ± 0.11, F(1, 53) = 37, p < 0.001).

We then turned to the fMRI data to understand how the brain represents and weighs up social and non-social information and how it facilitates (suppresses) the impact of the more (less) reliable information on choice. Notably, only in incongruent trials can we unambiguously infer what information (social or non-social) guided choice. Using a whole-brain general linear model (GLM) (GLM3 in STAR Methods), we divided our trials into three categories as follows: incongruent trials in the face condition when animals followed face direction, incongruent trials in the object condition when animal chose against the information provided by the face, and all congruent trials.

**Figure 3:**
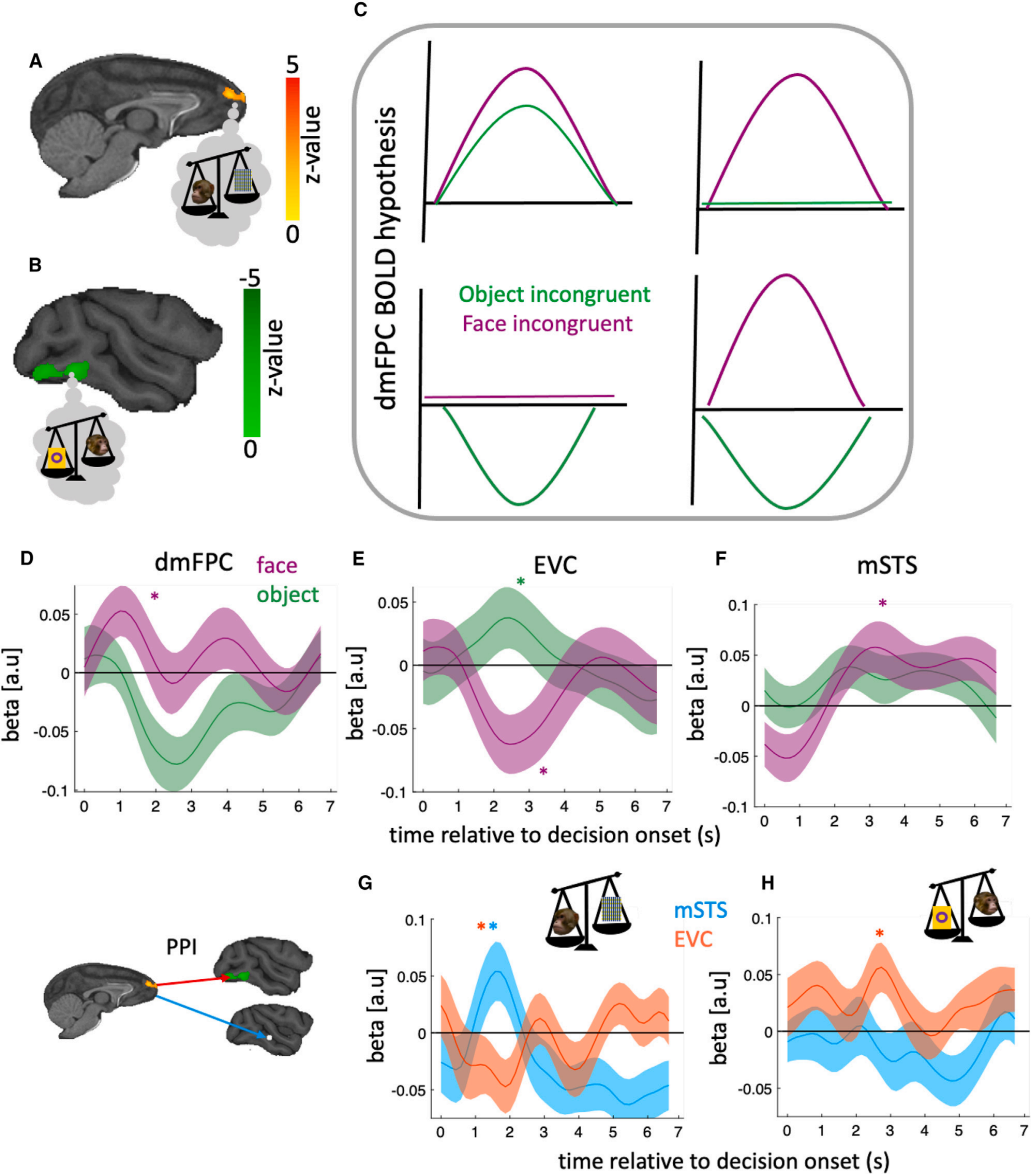
dmFPC involvement in our task (A and B) Whole-brain analysis showed that dmFPC peak Montreal Neurological Institute (MNI) coordinate [8 27 14] (A) and EVC peak MNI coordinate [2 −43 1] (B) were the only areas in which activity was significantly different between the face and the object conditions on incongruent trials. The activation maps were obtained by subtracting the incongruent trials in the face condition from the incongruent trials in the object condition. (C) The positive effect in dmFPC could be due to different possible patterns of activity. Top left, if dmFPC is positively correlated with incongruency in both conditions, we expect positive dmFPC response to incongruency in both conditions, but more so in the face condition. Such a pattern of activity would be consistent with dmFPC encoding incongruency. Top-right, if dmFPC was only critical in incongruent face trials, we expect positive correlation with incongruency in the face condition, but no significant relationship with incongruency in the object condition. This would suggest that dmFPC facilitates the impact of social information when it is reliable. Bottom-left, if dmFPC was only critical in incongruent object trials, we expect a negative correlation between dmFPC activity and incongruency in the object condition, but no significant relationship with incongruency in the face condition. This would suggest dmFPC suppresses the impact of object information when it is unreliable. Bottom-right, if dmFPC relationship with incongruency was different between the two conditions, we expect positive correlation with incongruency in the face condition and negative correlation in the object condition. This would suggest that dmFPC regulates the impact of social information according to its reliability—promoting social information in the face condition when it is reliable and suppressing the object information, which is attended by default, when it is unreliable. In addition, it would suggest that dmFPC suppresses social information when it is unreliable in the object condition. (D) In incongruent face trials, dmFPC activity increased (compared with congruent trials), whereas it decreased in incongruent object trials. (E) The opposite pattern was observed in EVC. (F) mSTS activity as a function of incongruency in each condition. (G) Functional connectivity between dmFPC and face and object related areas in each condition. (H) Same as (F) but in the object condition. In (D)–(F), y axis indicates $\beta_{1}$ in Equation 8. In (G)–(H), y axis indicates $\beta_{3}$ in Equation 9. In (D)–(H), the line and the shaded area represent mean and standard error of the mean (SEM), respectively. ^∗^$p_{FDR}$ < 0.05.

Activity in extrastriate visual cortex (EVC), including areas V4 and TEO (henceforth EVC), was significantly higher when monkeys were focusing on the visual objects themselves rather than on the oriented monkey face; it was more active in the incongruent object versus incongruent face condition (Figure 3B). This latter finding is consistent with previous demonstrations that this region is important when monkeys learn about and attend to visual objects.^26^^,^^27^^,^^28^^,^^29^ By contrast, however, we found that dorsomedial frontopolar cortex (dmFPC) was the only area that had significantly higher activity in the incongruent face versus incongruent object condition (Figure 3A; Table S1 for details). Notably, the difference in dmFPC activity between face and object conditions was specific to incongruent trials: there was no difference in dmFPC activity between the two conditions in congruent trials (Figure S2A).

The positive effect in dmFPC could arise because of distinct patterns of activity (Figures 3C–3F). To tease apart these possibilities, we performed additional time-course analyses of dmFPC activity (see STAR Methods). Since we had very few incongruent trials in the mixed condition, we restricted our analyses to face and object conditions. We ran separate regression models for face and object conditions. In these models, congruency (1 and 0 for incongruent and congruent trials, respectively) and reaction time were included as independent variables, and blood-oxygen-level-dependent (BOLD) signal as a dependent variable. We found that dmFPC activity was more positive in the incongruent face condition when the face was followed (Figure 3G, $p_{FDR}$ = 0.01, HBC), whereas it was more negative in the incongruent object condition when the face was not followed (Figure 3G, $p_{FDR}$ = 0.01, HBC). An approximately opposite effect was observed in EVC: it was negative in the incongruent face condition (Figure 3H, $p_{FDR}$ = 0.02, HBC), but not significantly different from zero in the incongruent object trials (Figure 3H, $p_{FDR}$ > 0.05, HBC). Finally, mSTS, the area that encoded face direction in experiment 1, was more active in the incongruent face condition (Figure 3I, $p_{FDR}$ = 0.01, HBC) but not in the incongruent object condition (Figure 3I, $p_{FDR}$ > 0.05, HBC), with no difference between the two conditions ($p_{FDR}$ > 0.05).

Next, we examined whether functional connectivity between dmFPC, on the one hand, and mSTS or EVC, on the other hand, varied as a function of experimental condition (face or object, see the Figure S2 legend for details). Here, we predicted dmFPC activity using incongruency as the psychological variable and mSTS activity as the physiological variable. Consistent with our prediction, in the face condition, dmFPC connectivity with mSTS increased in incongruent trials (Figure 3J, $p_{FDR}$ = 0.008, HBC), whereas its connectivity with EVC did not change (Figure 3J, $p_{FDR}$ > 0.05). In contrast, in the object condition, dmFPC-EVC connectivity increased in incongruent trials (Figure 3K, $p_{FDR}=0.04$, HBC), whereas FPC-mSTS connectivity was not different from zero (Figure 3K, $p_{FDR}$ > 0.05).

So far, our findings suggest that dmFPC facilitates the impact of social information on choice when it is reliable but limits its impact when it is unreliable, an interpretation that we return to in experiment 3, below. Notably, the “reliability assessment” role proposed for dmFPC contrasts with top-down control over sensory processing in the absence of ambiguity and a need to infer the reliability of an information source. Contrary to what may have been expected, ventral prearcuate cortex, an area associated with exerting top-down control over other sensory areas played little role in our task.^30^^,^^31^

We performed additional analyses that were time-locked to outcome rather than decision time to test whether dmFPC activity patterns were specific to the process of decision making. We found that all dmFPC patterns of activity were specific to decision time and were not observable at outcome time (Figure S2). We repeated these analyses on another area of the prefrontal cortex (PFC), 47/12o. Unlike the dmFPC activity pattern found in our task, which is specific to decision time, 47/12o is important for learning and outcome processing.^32^^,^^33^^,^^34^ In Figure S2, we show that 47/12o’s activity pattern is not specifically related to decision. Therefore, dmFPC activity is most likely related to decision making itself or computational processes on which decision making depends (Figure 2B).

### Experiment 3

Finally, we examined whether dmFPC was causally responsible for determining whether social information would guide decision making. The same three animals completed 12 more sessions consisting of face and object conditions (50 trials each) after receiving offline TUS or sham. In the active sessions, TUS was applied before behavioral testing to one of the three areas (dmFPC, mSTS, or EVC). In sham sessions, no stimulation was applied. Each animal completed four rounds of data collection in each of the four experimental conditions (STAR Methods).

**Figure 4:**
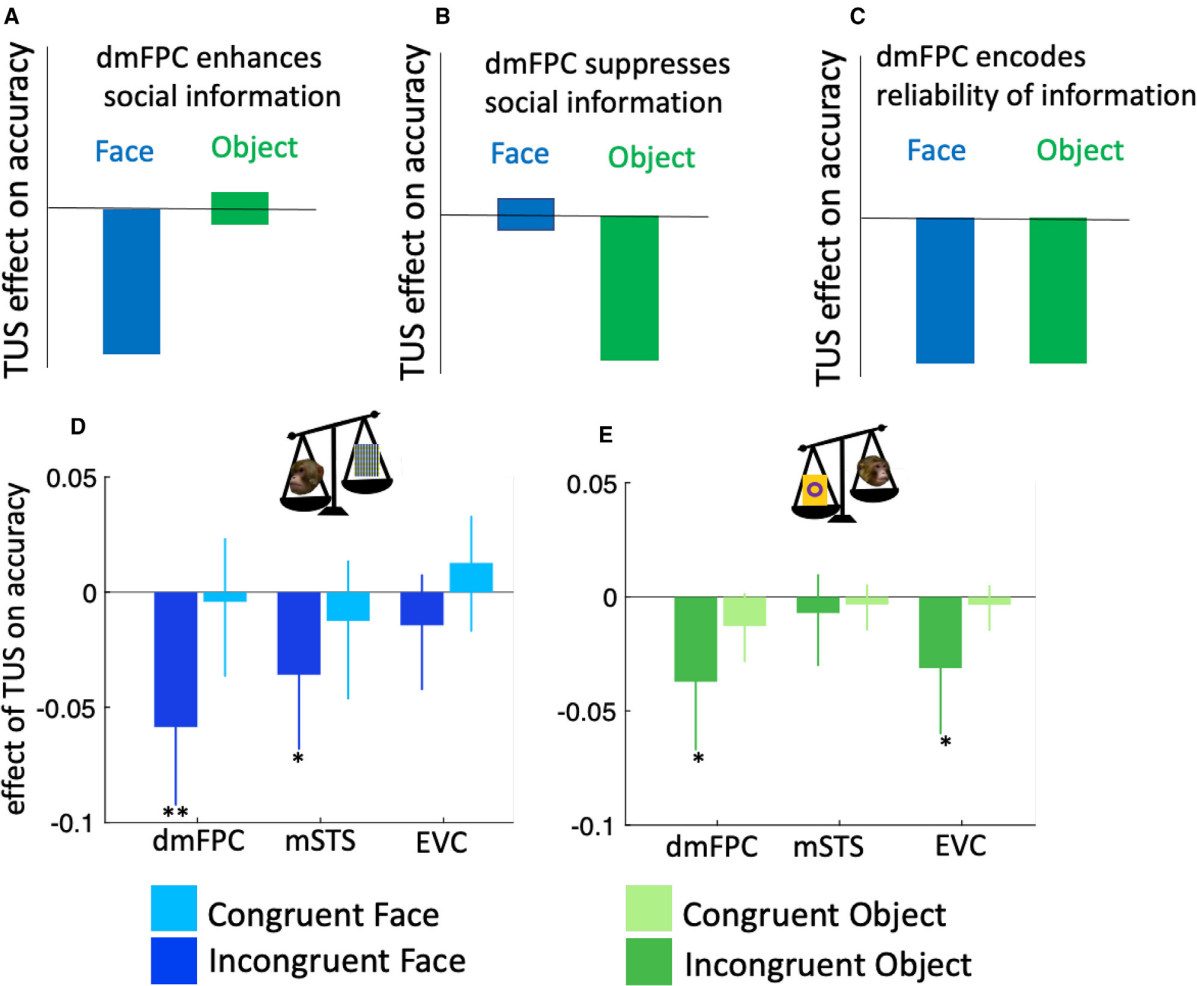
Result of TUS experiment (experiment 3) on different regions (A) If dmFPC only enhances social information, its disruption would impair performance in the face, but not in the object, condition. (B) If dmFPC is only important in suppressing social information, its disruption would impair performance only in the object condition. (C) If dmFPC regulates the impact of social information based on its reliability, its disruption should impair performance in both face and object conditions. (D and E) We used linear regression models to quantify the impact of TUS on animals’ accuracy in face (D) and object (E) conditions, separately for congruent and incongruent trials. The y axis indicates $\beta_{2}$ in Equation 3, which captures difference in accuracy in each stimulation condition against sham. In (D) and (E) error bars indicate 95% confidence interval. ∗ p <. 05, ∗∗ p <. 001

Different hypotheses about the role of dmFPC predict different deficits in behavior (Figures 4A–4C). We ran LMMs (LMM3 in STAR Methods), in which we directly compared each stimulation site with sham. The models were run separately for congruent and incongruent trials. We found that in the incongruent face condition, there was a significant reduction in accuracy with dmFPC-TUS compared with sham (Figure 4D; β ± 95% CI = −0.05 ± 0.03, F(1, 548) = 11, p < 0.001, HBC). We found a similar reduction in accuracy when comparing mSTS-TUS versus sham (Figure 4D; β ± 95% CI = −0.03 ± 0.03, F(1, 552) = 4.68, p = 0.03, HBC), whereas there was no effect of EVC-TUS on accuracy (Figure 4D; β ± 95% CI = −0.01 ± 0.02, F(1, 551) = 1, p = 0.31).

Critically, stimulation in no area affected accuracy in congruent trials (Table S2). We directly compared TUS effects on accuracy between congruent and incongruent trials. As incongruency has a negative effect on accuracy (Figure 2E), we compared reduction in accuracy in incongruent trials across areas. If TUS specifically affects incongruent trials, the negative effect of incongruency should be more pronounced during TUS compared with sham. We therefore added incongruency to our linear regression model and compared dmFPC-TUS versus sham. We found a significant negative interaction between TUS and incongruency (β ± 95% CI = −0.02 ± 0.02, F(1, 1,179) = 5, p = 0.02) meaning that compared with sham, dmFPC-TUS had a more negative impact on accuracy in incongruent than in congruent trials. This difference did not reach significance in the mSTS-TUS versus sham comparison (β ± 95% CI = −0.01 ± 0.02, F(1, 1,176) = 1.67, p = 0.19).

We tested our hypotheses in the object condition where we expected a reduction in accuracy following dmFPC and EVC-TUS. We found a significant reduction in accuracy in dmFPC versus sham (Figure 4E, β ± 95% CI = −0.06 ± 0.06, F(1, 584) = 5.5, p = 0.03, HBC) and in EVC versus sham (Figure 4E, β ± 95% CI = −0.06 ± 0.06, F(1, 584) = 4.7, p = 0.03, HBC), whereas, as expected, there was no effect in mSTS versus sham (Figure 4E, β ± 95% CI = −0.01 ± 0.07, F(1, 579) = 0.23, p = 0.62, HBC). We found that these effects were not significantly different between congruent and incongruent trials in the object condition. As before, we quantified this by asking whether reduction in accuracy in incongruent trials was more pronounced in dmFPC versus sham (β ± 95% CI = −0.01 ± 0.02, F(1, 1,182) = 0.77, p = 0.15) and EVC versus sham (β ± 95% CI = −0.01 ± 0.02, F(1, 1,161) = 3.12, p = 0.07). However, after combining face and object conditions, we found that the reduction in accuracy was significantly higher in incongruent compared with congruent trials following dmFPC-TUS (β ± 95% CI = −0.02 ± 0.01, F(1, 2,392) = 7.04, p = 0.008). On repeating LMM3 for congruent trials, we found no effect of TUS on accuracy (Table S2). Therefore, dmFPC-TUS specifically targeted incongruent, but not congruent, trials. Finally, we show that the impairment in the incongruent trials indicates that the effect of more reliable information in each condition was reduced following TUS (Table S3).

## Discussion

dmFPC evaluates and determines the impact of social information on choice by adjusting its activity according to the reliability of the social information. Its activity was responsive to a combination of congruency and reliability: it was highest when social information was reliable and incongruent to the non-social information and at its nadir when social information was unreliable and incongruent to the non-social information. Consistent with these findings, our TUS results showed that dmFPC-TUS led macaques to fail to be guided by social information when it was reliable but to be more likely to use it when it was unreliable. Temporoparietal junction (TPJ) and dorsomedial PFC (dmPFC)—the human homologs of mSTS and dmFPC, respectively—are prominent in many social tasks.^5^^,^^35^^,^^36^^,^^37^ It has been suggested that dmPFC in humans may be important for inferring others’ mental state.^38^^,^^39^ Others have suggested a more general role for this area in social cognition,^40^ whereas a recent study has suggested that dmPFC is involved in separating the currently relevant social information from the currently irrelevant ones.^37^ Our study shows that dmFPC in monkeys might play a similar role: separating reliable information from the unreliable. According to this view, dmFPC plays a general role in weighing information according to its relevance, regardless of whether the relevance is imposed by context. Such a function is broadly consistent with dmFPC’s tracking of the significance of alternative goals and facilitation of behavior switching.^41^^,^^42^ This can be tested in future studies using more general cue-combination tasks^43^ to develop computational theories about the broader role of frontal areas like dmFPC. Finally, it is likely that dmFPC is involved not only in weighing social information according to reliability but also in learning the reliability itself. However, our study was not designed to answer this question; our subjects performed the task inside the scanner after having already learned about the reliabilities of the different stimuli and, consequently, reached near optimal performance during pre-task training (Table S4).

It has been shown that the brain computes a weighted average (according to reliability) to combine different sources of information.^16^^,^^43^ Our study indicated that the information provided by sensory areas (such as mSTS and EVC) is processed and moderated by frontal areas. We found that dmFPC activity was modulated by a combination of the reliability and congruency of the social information: it was at its peak when the social information was reliable and incongruent to the non-social information, and its nadir when social information was unreliable and incongruent to the non-social information. Critically, this process of reliability-based weighting of information was achieved through the interaction between frontal and temporal-cortical areas. Our study provides a computational account for how such a reliability-based weighting of information is implemented in the brain, which might extend to non-social contexts as well.

Previous studies have shown that certain regions of the human and non-human primate brain encode and predict the actions of other individuals in their environment.^23^^,^^44^ The primary use of this predictive mechanism has been linked to theory of mind. Our study, however, suggests that such information regarding others can also be used to guide the observing animals’ own behaviors when it is more reliable than other information sources.

## STAR★Methods

### Key resources table

**Table:** 

REAGENT or RESOURCE	SOURCE	IDENTIFIER
**Experimental models: Organisms/strains**		

Macaca mulatta, 3 males, between x and y years old, between x and y kg, socially housed	MRC, Centre for Macaques	NCBITaxon:9544

**Deposited data**		

Raw and analysed data	This paper	https://github.com/alimahmoodia/Monkey-Social-Information-Use

**Software and algorithms**		

MATLAB R2020b	MathWorks	RRID:SCR_001622 https://www.mathworks.com/
FMRIB Software Library v5.0	FMRIB, WIN, Oxford, UK	RRID:SCR_002823 http://www.fmrib.ox.ac.uk/fsl/
Advanced Normalization Tools	Tustison and Avants^45^	N/A
Magnetic Resonance Comparative Anatomy Toolbox	Neuroecology Lab	https://github.com/neuroecology/MrCat
Brainsight	Rogue Research	https://www.rogue-research.com/

### Resource availability

#### Lead contact

Further information and requests for resources should be directed to and will be fulfilled by the lead contact, Ali Mahmoodi (ali.mahmoodi1367@gmail.com).

#### Materials availability

All data and scripts to replicate the behavioral results are available at https://github.com/alimahmoodia/Monkey-Social-Information-Use. All neuroimaging data are available upon request.

#### Ethical compliance

All procedures were conducted under licenses from the United Kingdom (UK) Home Office in accordance with the UK Animals (Scientific Procedures) Act 1986 and with the European Union guidelines (EU Directive 2010/63/EU).

### Method details

#### Behavioral training

Three male rhesus macaques (*Macaca mulatta*), aged 9–13 years and weighing 13–17 kg, were involved in the study. The animals, further referred to as T, V, and W, were kept on a 12 h light-dark cycle. MRI-compatible cranial implants (Rogue Research) were surgically implanted to prevent head movements. The macaques were sat in a sphinx position in an MRI-safe chair with their head fixed for the duration of training and testing, using a surgically implanted PEEK headpost (Rogue Research). Responses were recorded using custom-built infrared sensors. Animals had access to water 16–18 h on testing days and with free water access on non-testing days and were deprived of food for 6–8 h before each test or training session.

Stimuli were presented using the Psychophysics Toolbox extension in Matlab.^46^

The macaques were trained to perform binary choices in a computer-based task over the course of 8 months. Training took place in a mock scanner with pre-recorded MRI noise played throughout. First, the animals were trained to associate reward with two objects, one on either side of the screen, before learning the objects’ reward probabilities. Next, the animals were taught to follow the direction exhibited by different macaque faces presented on each trial in order to receive reward. The animals were then trained on the three experimental conditions (Experiment 2: fMRI).

#### Experiment 1: encoding of face direction and identity

In Experiment 1, we conducted a repetition suppression experiment to identify brains areas that encode other animals’ face directions. Repetition suppression is the reduction of neural activity due to repeated presentation of a stimulus, or a stimulus feature.^47^^,^^48^ Here, we looked for brain areas with reduced activity when the direction of two macaque faces presented in close succession was similar compared to when the direction of the second face was dissimilar to the first face. Similarly, we looked for brain areas with reduced activity when the identities of two macaque faces were the same in comparison to when they had different identities. Each monkey completed 10 fMRI sessions of 140 binary choice trials (30 sessions in total, 2 excluded because one animal missed more than 20% of the trials). At the beginning of each trial, a face appeared in the centre of the screen (henceforth, first face). This face could be either *neutral* (gazing forward) or *directed* (gazing left or right). The first face was followed by 500 milliseconds of a blank screen, after which a second, directed face appeared. The animals had 3 seconds from the appearance of the second face to indicate the side the face was gazing at using the infrared sensors. In trials where the monkeys responded prematurely, the trial was terminated and repeated. If the first face was directed, the face direction of the second face was identical to the direction of the first face. However, if the first face was neutral, then the second face was directed either left or right. Additionally, the two faces might have the same identity or different identities. In total, the stimuli comprised 5 different faces, making 5 (identity) by 3 (direction) sets from which faces were selected. We ensured a balanced design by having the same number of trials in each condition of our 2 (same or different direction) by 2 (same or different identity) design. Only the correct trials were included in the final analysis.

#### Experiment 2: impact of social information on choice

Experiment 2 included 16 fMRI sessions per subject (48 sessions in total), with each session comprising 150 binary choice trials. At the beginning of each trial, a neutral (i.e., gazing forward) macaque face appeared in the centre of a black screen, with an object on either side of the face. After 100ms, the face turned towards one of the objects, and the subjects had 10 seconds to choose one of the two objects by placing either their left or right hand on the corresponding infrared sensor. This decision was then followed by an action-outcome-delay of 3-5 seconds, after which the rewarded object was indicated on the screen and the monkeys either received 2 drops of juice or no juice. Each trial was separated by an inter-trial interval (ITI) of between 3-4 seconds.

Trials were divided into three conditions of 50 trials each. in the Face condition, the face had a reliability of 90%, i.e., the face direction indicated the rewarded object in 90% of the trials. Whereas the object had a reliability of 60%, i.e., one object would result in reward in 60% of the trials while the other would result in reward in 40% of the trials. In this condition, the monkey would receive the most reward if it always followed the face direction. Analogously, in the Object condition, the object had a reliability of 90% while the face had a reliability of 60%. Here, the monkey would receive the most reward if it always chose the most reliable object. In the Mixed condition, both the face and the object had a reliability of 90%, so the monkey could choose whether to follow the face direction or to choose the more reliable of the two objects to obtain the most reward. The proportion of incongruent trials in different conditions were as follows: 39% in the Object condition, 37% in the Face condition, and 17% in the Mixed condition.

#### Experiment 3: causal manipulation using TUS

The transcranial ultrasound stimulation (TUS) sessions comprised four conditions: three active stimulation sites (the FPC, mSTS, and EVC) and one Sham condition. Each condition was repeated four times, making 16 sessions per animal in total, with the order of sites targeted pseudo-randomised for each animal. There was at least a 24 h gap between each stimulation session to avoid any carry-over effect.

Following stimulation, the monkeys were moved to a separate room for their behavioral session. Each behavioral session comprised 100 trials divided into two condition blocks. The two conditions were the *Face* condition and the *Object* condition, differing from the conditions in experiment 2 only by the action-outcome-delay (300ms). Each trial was followed by an inter-trial interval of 3 to 5 seconds. Because animal T and V showed side biases for this experiment, they were forced to press left and right sensors for a drop of juice every seven trials to minimise their side bias.

#### fMRI data acquisition and processing

MRI data were collected using a 15-coil 3T horizontal bore MRI scanner. Functional data were acquired using a gradient-echo T2∗ echo planar imaging (EPI) sequence with a 1.25 × 1.25 × 1.25 mm resolution, repetition time (TR) 1.48 s, echo time (TE) 30 ms, flip angle 70°, multiband acceleration factor 2 and GRAPA acceleration factor 2. °. T1-weighted MP-RAGE structural images with a 0.5 x 0.5 x 0.5 mm resolution, TR 2.06 s, TE 4.04 ms, were acquired during a separate session under anaesthesia.

T2^∗^ EPI images acquired during the experimental task were reconstructed using a custom offline reconstruction script, using techniques described previously.^49^ Preprocessing of structural and functional images involved tools from the FMRIB Software Library (FSL),^50^ MATLAB (R2022a, Mathworks), Advanced Normalisation Tools (ANTs; http://stnava.github.io/ANTs),^45^ and the Magnetic Resonance Comparative Anatomy Toolbox (MrCat; http://github.com/neuroecology/MrCat).

MP-RAGE images were iteratively pre-processed using a macaque-optimised pipeline using FSL’s brain-extraction tool (BET), RF bias-field correction, and linear and non-linear registration (FLIRT and FNIRT) to the *Macaca mulatta* McLaren F99 template implemented in MrCat. The pre-processed structural images of the three animals were then combined to create a group template image. This was accomplished using tools from ANTs as implemented in MrCat, by iteratively registering the initial structural MRI to F99 space, group averaging, and registration of the new group template.

For each session, to correct for distortions of the magnetic field due to motion, each slice of the functional image was linearly and non-linearly registered to a reference based on the EPI volumes from the same timeseries with the least distortion. The aligned and distortion-corrected image for each session was non-linearly registered to the high-resolution structural MRI reference of each subject, which was then registered to the group template using ANTs. EPI images were spatially smoothed (3 mm FWHM) and temporally high pass filtered (cut-off 100 s).

#### TUS protocol

Transcranial ultrasound stimulation (TUS) was performed using a four-element annular array transducer (NeuroFUS (CTX-250, 64mm active diameter, Brainbox Ltd, Cardiff, UK) combined with a programmable amplifier (Transducer Power Output System, TPO-105, Brainbox Ltd, Cardiff, UK). The transducer was paired with a transparent coupling cone filled with degassed water and sealed with a latex membrane. The resonance frequency of the ultrasonic wave was set to 250kHz. The stimulation comprised 30ms bursts of ultrasound), for a total stimulation duration of 40s. The stimulation protocol followed previously established protocols in macaques,^51^^,^^52^ delivered through a Transducer Power Output (TPO) system (Sonic Concepts). The spatial-peak pulse-average intensity (I_SPPA_) in water (free field) was 60.0 W/cm^2^. This intensity is attenuated when passing through the skull.

At the beginning of each stimulation session the animal’s skull was shaved and a conductive gel (SignaGel Electrode; Parker Laboratories Inc.) was applied to the skin. The water-filled coupling cone and the gel were used to ensure ultrasonic coupling between the transducer and the animal’s head. Next, the ultrasound transducer / coupling cone montage was placed on the skull and a Brainsight Neuronavigation System (Rogue Research, Montreal, CA) was used to position the montage so that the focal spot was centered on the targeted brain region. All targets were sonicated bilaterally for 80 s in total, with 40 s of stimulation applied to a target in each hemisphere (except for FPC which was only stimulated for 40s as the target was on the midline). Sonication of the target in one hemisphere was immediately followed by sonication of a homologous target in the contralateral hemisphere. Hemispheres were sonicated in a pseudo-random order. After stimulation, monkeys were immediately moved to a testing room for behavioral data collection.

There were four stimulation conditions: the frontal polar cortex (FPC) [0, 27, 9] the medial superior temporal sulcus (mSTS) [25.2, -15.6, -0.5] and [-22.0, -14.6, -4.6], the extrastriate visual cortex (EVC) [23, -35, -4] and [-23, -35, -4], and Sham. Note that the precise EVC coordinate that we reported in our fMRI results was not accessible to the TUS coil. We therefore used the coordinate mentioned above which was associated with qualitatively identical activity patterns to those seen at the area of peak change reported in the fMRI Results sections. The three active stimulation sites were projected onto individual T_1_-weighted MRI brain scans using the Brainsight Neuronavigation System to inform accurate online positioning of the transducer over each target region during stimulation. The Sham sessions, included as a non-stimulation passive control, were identical to the active stimulation sessions, except that the sonification was not applied. During the sham sessions, the ultrasound transducer was randomly placed over one of the target sites.

### Quantification and statistical analysis

#### Behavioral data analysis

##### Linear mixed effect models

In all linear regression models (see below for details), we assessed the statistical significance of model parameters by F-statistics and Satterthwaite’s approximation for degrees of freedom.

##### LMM1

To investigate the effect of the social and object information on choice in experiment 2, we conducted a linear mixed effect model as follow:

(Equation 1) $${choice}_{t}=\beta_{1s}+\beta_{2s}\times face\;direction_{t}+\beta_{3s}\times object\;direction_{t}+\beta_{4s}face\;direction_{t}\times object\;direction_{t}$$

Where ${choice}_{t}$ indicates animals’ choice on trial t and was set to 1 if left was selected and 2 otherwise. $face\;direction_{t}$and $object\;direction_{t}$ indicate the direction of the face and the side of the object with the higher probability of reward in trial t (1 for left and 2 for right), respectively. The intercept ($\beta_{1s})$and all slopes ($\beta_{ks}$) were allowed to vary across sessions (denoted by s) by including random effects of the form $\beta_{ks}=\beta_{k0}+b_{ks}$ where $b_{ks}∼N\left(0,\sigma_{k}^{2}\right)$.

##### LMM2

To investigate the effect of incongruency on accuracy in experiment 2, we conducted a linear mixed effect model as follow:

(Equation 2) $${accuracy}_{t}=\beta_{1s}+\beta_{2s}\times congruency_{t}$$

Where ${accuracy}_{t}$ indicates animals’ accuracy in trial t (0 and 1 for wrong and correct responses, respectively), and $congruency_{t}$ indicates congruency in trial t and was set to 0 for a congruent trial and to 1 for an incongruent trial. The intercept ($\beta_{1s})$and $\beta_{2s}$ were allowed to vary across sessions (denoted by s) by including random effects of the form $\beta_{ks}=\beta_{k0}+b_{ks}$ where $b_{ks}∼N\left(0,\sigma_{k}^{2}\right)$.

##### LMM3

For analysis of experiment 3 (TUS experiment), for each of the two trial types (congruent and incongruent), we ran a linear mixed-effects model to investigate the effect of stimulation on accuracy. To this end we compared each active stimulation site (FPC, mSTS, EVC) versus sham separately for congruent and incongruent trials and for each condition (Face and Object) as follows:

(Equation 3) $${accuracy}_{t}=\beta_{1s}+\beta_{2s}\times {ROI}_{t}$$

Where ${accuracy}_{t}$ indicates animals’ accuracy in trial t (0 and 1 for wrong and correct responses, respectively), and ${ROI}_{t}$indicates the ROI/condition of stimulation in trial t and was set to 0 for all trials in the Sham sessions and to 1 for all trials in the active stimulation sessions. The intercept ($\beta_{1s})$and $\beta_{2s}$ were allowed to vary across sessions (denoted by s) by including random effects of the form $\beta_{ks}=\beta_{k0}+b_{ks}$ where $b_{ks}∼N\left(0,\sigma_{k}^{2}\right)$.

Where the Hessian Matrix of the model was not positive definite, potentially due to overparameterization of the model, we changed all the mixed effect variables in Equation 3 to fixed effect to ascertain a positive definite Hessian matrix which is indispensable for reliable beta estimation.

We used the same model (Equation 3) to compare active sessions against each other. For this purpose, we included two active stimulation sessions (e.g., FPC and EVC) in the model instead of one active stimulation session and one Sham session.

##### LMM4

For each of the two trial types (congruent and incongruent), we ran a linear mixed-effects model to investigate the effect of stimulation on the contribution of social information on choice. To this end we compared each active stimulation site (FPC, mSTS, EVC) versus sham separately for congruent and incongruent trials and for each condition (Face and Object) as follows:

(Equation 4) $${choice}_{t}=\beta_{1s}+\beta_{2s}\times face\;direction_{t}+\beta_{3s}\times {ROI}_{t}+\beta_{4s}\times face\;direction_{t}\times {ROI}_{t}$$

Where ${choice}_{t}$ indicates animals’ choice on trial t and was set to 1 if left was selected and 2 otherwise. $face\;direction_{t}$ indicates the direction of the face in trial t (1 for left and 2 otherwise).${ROI}_{t}$ indicates ROI/condition of stimulation in trial t and was set to 0 for all trials in the Sham sessions and to 1 for all trials in the active stimulation sessions. The intercept ($\beta_{1s})$and all slopes ($\beta_{ks}$) were allowed to vary across sessions (denoted by s) by including random effects of the form $\beta_{ks}=\beta_{k0}+b_{ks}$ where $b_{ks}∼N\left(0,\sigma_{k}^{2}\right)$.

To investigate the effect of object information on choice, we replaced $face\;direction_{t}$with $object\;direction_{t}$ in Equation 4, where $object\;direction_{t}$indicates the direction of the object with the higher probability of reward in trial t (1 for left and 2 for right).

In all linear mixed-effect models, the model parameters were estimated using Maximum Likelihood method of fitting. After running each model, we confirmed the normality assumption of the model by running a Kolmogorov-Smirnov test.

We initially included all variables as random effects, but if the Hessian Matrix of the model was not positive definite, potentially due to overparameterization of the model, we changed the mixed effect variables to fixed effect to ascertain a positive definite Hessian matrix which is indispensable for reliable estimation.

In all models that we conducted for the Face and Object conditions we always compared the full model (which included both face and object direction and their interaction) with a simpler model which only contained the more reliable information on that block using likelihood ratio test. In all cases, the full model provided the best model fit.

#### fMRI data analysis

fMRI data were analysed using FSL. The whole-brain functional data was analysed in FEAT (FMRI Expert Analysis Tool) Version 6.00, part of FSL, using a univariate general linear model (GLM) approach. At the first level, FEAT computed contrasts of parameter estimates, and variance estimates for each contrast. These estimates were then transformed into standard space using ANTs. Higher-level analyses utilised FLAME 1+2 (FMRIB’s Local Analysis of Mixed Effects, stage 1 and 2) and treated sessions as random effects.

In experiment 1, two sessions were excluded from the fMRI analyses because monkey V did not finish the sessions. Therefore, experiment 1 comprised 28 total sessions. In experiment 2, two sessions were excluded from the analyses (one session from T and one session from W) due to technical issues. Thus, experiment 2 comprised 46 sessions.

#### GLMs 1 & 2 (whole brain analysis for experiment 1)

For the whole-brain analysis of experiment 1, we conducted the following univariate GLMs. GLM1 was conducted to test for brain areas that encoded face direction:

(Equation 5) $$BOLD=\beta_{1}face+\beta_{2}dsimFace+\beta_{3}simFace+\beta_{4}RT+\beta_{5}leftResp+\beta_{6}rightResp+\beta_{7}outcome+\beta_{8}binOutcome+\beta_{9}leftUnconv+\beta_{10}rightUnconv+\beta_{11}juice+\beta_{12}fchInvalid+\beta_{13}schInvalid$$

Regressors 1-8 and 12-13 were HRF-convolved with a gamma function (mean lag 3s, standard deviation 1.5s). Here, BOLD is a t x 1 (t time samples) column vector containing the time series data for a given voxel. face represents the onset of the first face (constant regressor coded as 1). In the face direction analysis, dsimFace and simFace represent the decision onset when the second face direction was dissimilar or similar to the first face, respectively. RT represents the reaction time in all trials, time-locked to decision onset and z-scored. leftResp and rightResp are regressors aligned to the decision onset in trials in which a left- or right-hand movement was recorded, respectively. outcome is the main effect of outcome (constant regressor encoded as 1) time-locked to the onset of feedback (i.e., juice). binOutcome ∈ (1,0) represents the effects of the outcome and was assigned 1 if the macaque received juice reward, and 0 if they answered incorrectly. It was time-locked to the onset of feedback. leftUnconv, rightUnconv, and juice, are unconvolved regressors that model signal distortions in the magnetic field due to movement, and so are not convolved with HRF. fchInvalid and schInvalid model the onset of the first and second face on all invalid trials. A trial was labelled invalid if the animal failed to respond at all or the response was slower than 1.5 seconds for animals V and W or 2.5 seconds for animal T (because this animal had a slower reaction time). In addition, task-unrelated confound regressors were included in the model to further reduce variance and noise in the BOLD signal. These regressors comprised 13 parametric PCA components describing the warping from each volume to the mean reference image when correcting for distortion in the magnetic field due to motion.

GLM2 was conducted to test for brain areas that encoded face identity. This GLM differed from GLM1 in only two regressors: regressors 2 and 3 modelled trials in which the second face was similar or dissimilar to the first face in terms of identity (instead of face direction).

The duration of all convolved regressors was a boxcar of 500ms. The duration of all unconvolved regressors was one TR.

#### GLM 3 (whole brain analysis for experiment 2)

For the whole brain analysis of experiment 2, we conducted the following GLM:

(Equation 6) $$BOLD=\beta_{1}object+\beta_{2}face+\beta_{3}congruent+\beta_{4}noResp+\beta_{5}RTvalid+\beta_{6}RTinvalid+\beta_{7}leftResp+\beta_{8}rightResp+\beta_{9}binOutcomeObject+\beta_{10}binOutcomeFace+\beta_{11}binOutcomeCong+\beta_{12}leftUnconv+\beta_{13}rightUnconv+\beta_{14}juice$$

Regressors 1-11 were HRF-convolved with a gamma function (mean lag 3 s, standard deviation 1.5s). In Equation 6, BOLD is a t x 1 (t time samples) column vector containing the time series data for a given voxel. object and face represent the main effects at the time of decision in incongruent trials where the object or face was followed, respectively, while congruent represents the main effects at the time of decision in congruent trials. noResp represents trials where no response was received. RTvalid and RTinvalid represent the reaction times in valid and invalid trials, respectively, where invalid trials are trials in which the animals took > 10 seconds to respond. These regressors were time-locked to decision onset and z-scored. leftResp and rightResp are regressors aligned to the decision onset of trials in which a left- or right-hand movement was recorded. binOutcomeObject, binOutcomeFace, and binOutcomeCong are binary {1,0} regressors representing main effects at the time of outcome in incongruent object and face trials, and congruent trials, respectively. The regressor was coded 1 if the animal received reward and 0 if they did not. The final three regressors (leftUnconv, rightUnconv, juice) were unconvolved. These conditions caused changes in the magnetic field due to movement, and thus the regressors model the instant signal distortions rather than neural activity. As before, 13 task-unrelated confound regressors were also added to the model. For all the convolved regressors, we used a boxcar of 1 second in length. The duration of outcome related regressors was 2 seconds. For the duration of non-convolved regressors we used a boxcar of one TR in length.

#### Activity time course analysis

For each scan session, we regressed out variation due to head motion, and up-sampled the BOLD time course to a resolution of 10 samples per TR. For each trial, we extracted activity time-locked to the onset of each event of interest up to 7 seconds after the onset of the event. We used linear regression to predict the ROI activity time courses. More specifically, we applied a linear regression to each time point and then, by concatenating beta-weights across time points and averaging across voxels, created a beta-weight time course for each predictor of a regression model. We performed this step separately for each session and pooled beta-weight time courses across sessions and subjects for visualisation. We then computed the average across all sessions as the observed beta weights. The significance of each time points from zero was determined using a permutation test. For a given time point, separately for each session, we shuffled the trial labels (preserving the number of trials) and computed the regression beta for each time point and session. We repeated this procedure 1,000 times to establish the null distribution. After each repetition, we computed the average across all sessions. We subsequently compared the shuffled beta weights to the observed beta weights to report the *p*-value. We computed the *p*-value from the onset of each event until the monkey hemodynamic response function reaches its peak (i.e., 3 seconds after the onset of each event). We then calculated the FDR-corrected *p*-values and reported the FDR-corrected *p*-values in the main text.

We used Equation 7 below for performing time course analyses pertaining to repetition suppression analysis, Figures 1B–1D:

(Equation 7) $$BOLD_{t}=\beta_{0}+\beta_{1}\times tria{l\;type}_{t}+\beta_{2}\times {RT}_{t}$$

Where $BOLD_{t}$ indicates the BOLD response on trial t. $Tria{l\;type}_{t}$ indicates whether the direction of the two faces were similar or dissimilar on trial t. It was set to 1 if the direction of the two faces in trial t were dissimilar and 0 otherwise.

We used Equation 8 below for performing time course analyses pertaining to Figures 3D–3F:

(Equation 8) $$BOLD_{t}=\beta_{0}+\beta_{1}\times tria{l\;type}_{t}+\beta_{2}\times {RT}_{t}$$

Where $BOLD_{t}$ indicates the BOLD response on trial t. $Tria{l\;type}_{t}$ indicates whether the trial was incongruent or congruent on trial t. It was set to 1 if the trial was incongruent and 0 otherwise.

We used Equation 9 below for PPI pertaining to Figures 3G and 3H:

$${dmFPC\;BOLD}_{t}=\beta_{0}+\beta_{1}\times {incongruency}_{t}+\beta_{2}\times {physio}_{t}+$$

(Equation 9) $$\beta_{3}\times {incongruency}_{t}\times {physio}_{t}$$

Where ${dmFPC\;BOLD}_{t}$ is dmFPC BOLD response on trial t. ${Incongruency}_{t}$ indicates whether a trial was incongruent and was set to 1 if so and 0 otherwise. ${Physio}_{t}$ indicates BOLD response of the physiological variable on trial and was extracted from mSTS or EVC for creating Figures 3G and 3H, respectively.

#### Creating Region of Interests (ROIs) for the time course analysis

The coordinate for creating mSTS, lateral mSTS and AM were taken from a previous study^23^ as follows (all coordinates in F99 standard space [x, y, z]): mSTS (25.2, -15.6, -0.5; -22.0, -14.6, -4.6), lateral mSTS (30.7; -16.1, 4.5; -27.6, -17.2, 2.5), AM (22.1, -1.5, -16.3; -20.5, -07, -14.7). The ROIs for FPC was created by defining a spherical mask (r=2mm) centred approximately around the centre of the cluster (F99 standard space [x, y, z] 0, 27, 12) which was significantly positive in incongruent Face versus Object condition obtained in the whole brain analysis using GLM3. The ROIs for EVC were created by defining a spherical mask (r=2mm) centred approximately around the centre of the cluster (F99 standard space [x, y, z] 23, -35, -4) which was significantly negative in the incongruent Face versus Object condition obtained in the whole brain analysis employing GLM3.

## Data Availability

All data and scripts to replicate the behavioral results are available at https://github.com/alimahmoodia/Monkey-Social-Information-Use. All neuroimaging data are available upon request.

## Supplemental information

Document S1. Figures S1 and S2, Tables S1–S4, and supplemental references

Document S2. Article plus supplemental information
