# Supplementary material for: A frontopolar-temporal circuit determines the impact of social information in macaque decision making
Source: Neuron. 2024 Jan 3;112(1):84–92.e6. doi: 10.1016/j.neuron.2023.09.035 (PMC10914637; doi:10.1016/j.neuron.2023.09.035)
Supplement: Document S1. Figures S1 and S2, Tables S1–S4, and supplemental references [file mmc1.pdf]

Neuron, Volume *112*

Supplemental information

A frontopolar-temporal circuit determines the impact of social information in macaque decision making

Ali Mahmoodi, Caroline Harbison, Alessandro Bongioanni, Andrew Emberton, Lea Roumazeilles, Jerome Sallet, Nima Khalighinejad, and Matthew F.S. Rushworth

Supplementary Information

A frontopolar-temporal circuit determines the impact of social information in macaque decision-making

Primary Supplemental PDF:

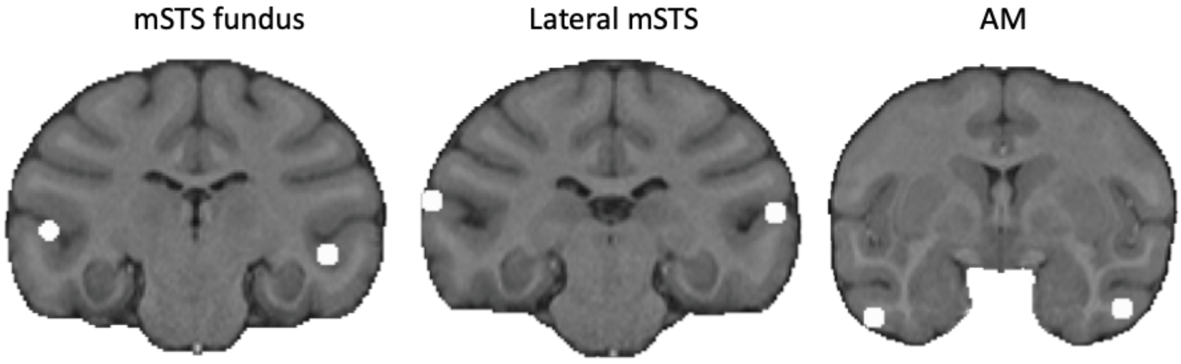

Figure S1: ***ROIs which were considered for experiment 1, related to Figure 1.*** **a** Coronal view of the three ROIs that we investigated for experiment 1. F99 standard space x, y, z coordinates for fundus mSTS: [25.2, -15.6, -0.5]; [-22.0, -14.6, -4.6], for lateral mSTS [30.7; -16.1, 4.5]; [-27.6, -17.2, 2.5] and for AM [22.1, -1.5, -16.3]; [-20.5, -.7, -14.7]. To analyse the data (**Repetition suppression analyses**), We optimised our design to exploit repetition suppression (a reduction in neural activity due to repeated presentation of a stimulus feature), to find brain areas encoding face direction by looking for areas in which activity was reduced when the second face direction was the same as, as opposed to different to, that of the first face. For this purpose, we ran a time course analysis at the time of the second face in which we regressed blood oxygen level dependent (BOLD) response as dependent variable onto face "direction similarity" and reaction time (as a confound variable) as independent variables. For each trial, direction similarity was set to 0 if the first and the second faces were gazing in the same direction, and to 1 otherwise. Therefore, if an area responds positively to this regressor, it means that it is exhibiting face direction-dependent repetition suppression. As explained in the methods of experiment 1, in addition to face direction, the two faces might have the same identity or different identities. In total, the stimuli comprised 5 different faces, making 5 (identity) by 3 (direction) sets from which faces were selected. We ran whole-brain GLMs to identify brain regions that showed significant repetition-suppression to face direction or face identity (see GLM1 and GLM2 in Methods). No area was significantly modulated by face direction at the whole brain level. One area, in posterior STS, was significantly modulated by face identity at the whole brain level, MNI coordinate [-22.6 -21.1 7.04], peak z-value 3.5, cluster size: 2043 (all coordinates correspond to the F99 atlas[1]).

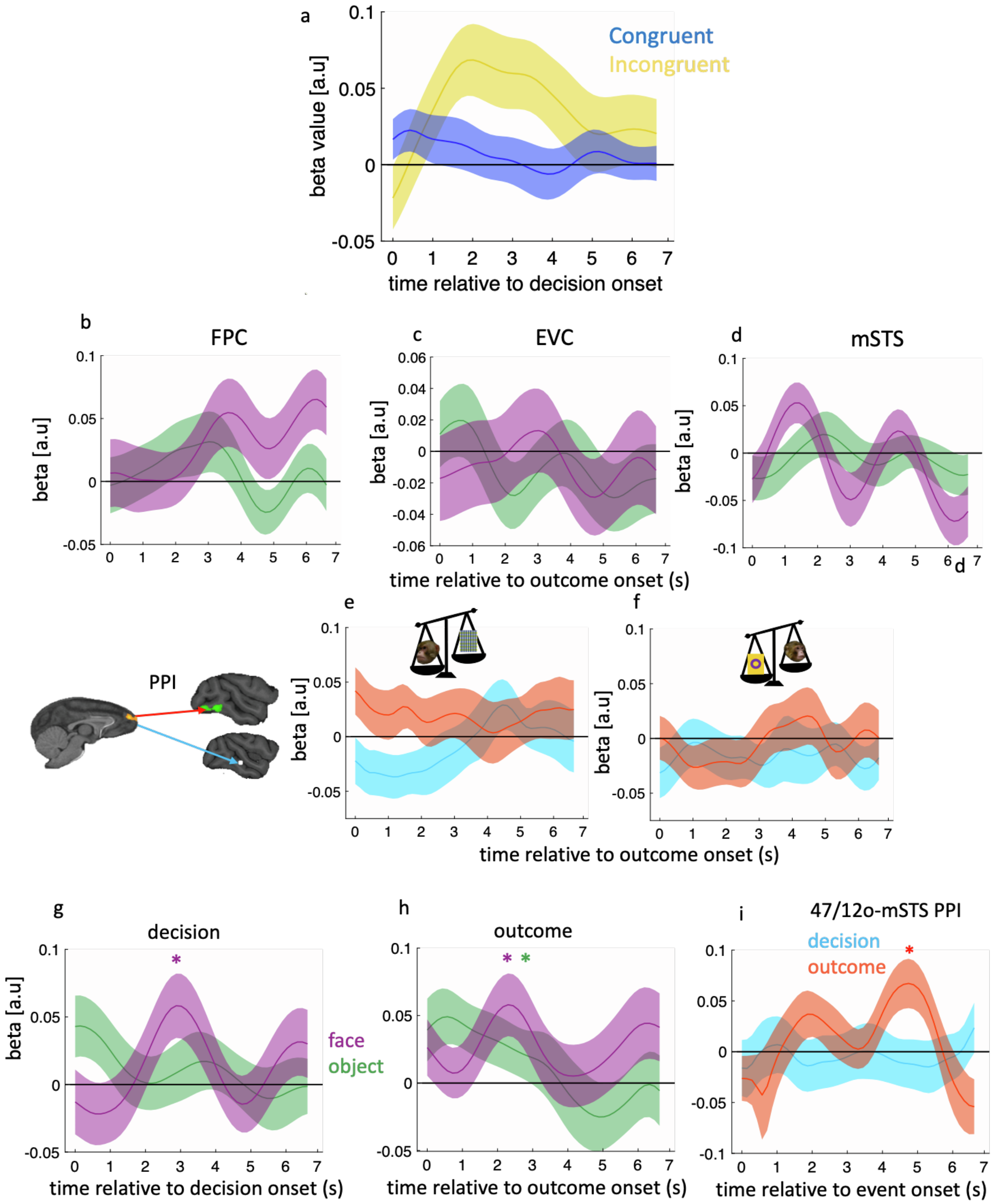

Figure S2: **Specificity of dmFPC activity incongruent trials at decision time, related to Figure 3. a** In experiment 2, We investigated the difference between the Face and Object conditions separately for the congruent and incongruent trials. To this aim, we ran a regression model in which we regressed dmFPC BOLD (as the dependent variable) against condition (coded as 1 for Face and 0 for Object, respectively) and reaction time. We repeated this procedure separately for the congruent (blue curve) and incongruent (yellow curve) trials. Consistent with our whole brain GLM in the main text (GLM3), we found that in dmFPC activity was significantly higher in the Face versus Object condition, but only in incongruent trials (W=765, p=.01), but not in congruent trials (W=639, p=.28). ***b-f*** Analogous to Figure 3 in the main text but at outcome time. dmFPC, EVC, and mSTS activity at outcome time and their functional

connectivity with other areas are different from decision time. None of the areas respond to incongruency at outcome time (b, c, d). In addition, there is no functional connectivity between dmFPC and EVC or dmFPC and mSTS neither in Face (e) nor in Object (f) condition at the time of outcome. ***g-i*** To investigate the specificity of the dmFPC activity and functional connectivity pattern that we observed, we repeated our analysis in another area of the frontal cortex, area 47/12o. Given the importance of this area in learning and credit assignment[2–7], we expected 47/12o's role, unlike dmFPC's, to be more specific to outcome time as opposed to decision time. We therefore expected its activity to differ from what we had observed in dmFPC. We extracted 47/12o's time course at both decision and outcome times and regressed it against incongruency. At decision time, 47/12o activity was significantly above zero in the incongruent Face condition (panel g, W=720, p=.04, HBC), but not Object condition (panel g, W=698, p=.08, HBC) and its activity in the incongruent trials was significantly higher in the Face than the Object condition (W=771, p=.01). Comparing its activity with dmFPC, we found that 47/12o's activity arose approximately 2s later, but its peak level of activity was not significantly different from dmFPC at decision time in the Face condition (W=521, p=.83), while they were significantly different in the object condition (W=746, p=.02). At the time of outcome, the 47/12o response to incongruency was significantly positive in both Face and Object conditions (panel h, Face W=779, p=.009, Object W=716, p=.05). However, its response to incongruency was higher in the Face compared to the Object condition (W=780, p=.008). In addition, the activity of 47/12o at outcome time in the Face condition (panel h) was significantly higher than dmFPC at outcome time (W=305, p=.01). To understand how it played this role, just as we had done for dmFPC, we examined 47/12o's functional connectivity (as seed region) with mSTS (as physiological variable) with incongruency as the psychological variable. Given the higher response of 47/12o to the Face condition and mSTS involvement in encoding face direction, we restricted analysis to the Face condition. Unlike for dmFPC-mSTS, there was no change in connectivity between 47/12o and mSTS at decision time (panel i, blue curve, W=320, p=.99, HBC) but their connectivity significantly increased at outcome time (panel i, red curve, W=749, p=.04, HBC). Finally, connectivity between dmFPC-mSTS was significantly higher than 47/12o-mSTS at decision time (W=273, p=.003), while there was a trend toward the opposite direction at the outcome time (W=399, p=.12).
To perform the ***Functional connectivity analyses,*** we performed the following procedures. Functional connectivity between two brain regions can be assessed using psychophysiological interaction (PPI). In a PPI analysis, activity of a brain region (seed region) is predicted using the interaction between activity of another brain region (physiological variable) and a psychological variable, while controlling for both main effects (physiological and psychological variables). An issue when doing functional connectivity analysis is to what degree the areas involved in the analysis are anatomically connected to each other. Most anatomical connectivity studies regarding mSTS have emphasized connections with the dorsal bank of the STS rather than the fundus and adjacent ventral bank that is the focus of the current study. However, because at each rostrocaudal level of the STS there are strong connections between each STS area and the laterally and medially adjacent STS areas[8], there is a two stage corticocortical route between dmFPC and mSTS. Similarly, there are no direct connections between dmFPC and the extrastriate areas, such as areas V4/TEO at the centre of the EVC region. EVC was linked to the guidance of decision making by visual object identity as opposed to faces. There are, however, other two-stage corticocortical routes known to influence activity in EVC that might run via ventrolateral prefrontal cortex[9–12]. Intriguingly, however, there are also subcortical routes by which dmFPC might modulate mSTS and EVC activity, for

example via thalamic nuclei such as the pulvinar, where face-responsive neurons have also been reported[13], which is connected with dmFPC, mSTS and EVC[14,15], and which is known to modulate visual activity[14,16].

			Peak MNI coordinates		
**Activation**	**Cluster size**	**Z**	**x**	**y**	**z**
		**Face minus object at decision time**			
Positive	2155	4.52	8.55	27.7	14.1
Negative	6206	4.05	2.01	-43.3	2.01
	3802	4.69	14.6	-41.7	-9.05
		**Face minus object at outcome time**			
Positive	21814	8.08	14.1	16.1	16.1
	2175	4.32	27.7	1.51	-6.04
Negative	15535	5.96	-27.7	0.5	12.6
	5261	4.02	-4.02	25.7	2.52
	4449	3.93	14.1	-1.51	0
	2880	4.39	-3.52	-12.1	-1.51
	1691	3.74	26.2	-6.54	10.1
		**Incongruent minus congruent trials at decision time**			
Positive	2236	3.49	-1.51	-25.7	4.02
		**Incongruent minus congruent trials at outcome time**			
Positive	11541	5.97	23.1	-3.52	14.6
	9086	5.47	-23.6	-2.01	9.05
Negative	1744	3.71	-1.01	16.1	1.51

Table S1: Whole brain results obtained from GLM3, related to Figure 3.

ROI	Condition	Beta±CI	F statistic	P-value
dmFPC vs Sham	Face	-.004±.06	.06	.80
STS vs Sham	Face	-.01±.07	.51	.47
EVC vs Sham	Face	.01±.06	.70	.40
dmFPC vs Sham	Object	-.01±.03	2.54	.11
STS vs Sham	Object	-.003±.02	.32	.57
EVC vs Sham	Object	-.003±.02	.34	.56

Table S2: TUS effect on congruent trials, related to Figure 4. Effect of TUS on congruent trials in the Face and Object conditions obtained by running LMM3. All p-values uncorrected.

ROI	Condition	Coefficient	Beta±CI	F statistic	P value
mSTS	Object	Object	.49±.07	690.08	<.001
		Face	.01±.05	.99	.32
	Face	Object	.01±.12	.15	.70
		Face	.89±.07	2411.70	<.001
dmFPC	Object	Object	.49±.12	250.21	<.001
		Face	.08±.08	17.11	<.001
	Face	Object	.04±.02	.52	.47
		Face	.80±.12	645.13	<.001
EVC	Object	Object	.47±.08	517.31	<.001
		Face	.04±.05	9.42	.002
	Face	Object	.02±.09	.95	.33
		Face	.94±.06	4431.00	<.001
Sham	Object	Object	.47±.05	1384.80	<.001
		Face	.01±.03	2.39	.12
	Face	Object	-.01±.10	.19	.67
		Face	.94±.06	3789.70	<.001

Table S3: Effect of face and object information on choice for Face and Object conditions, following each TUS condition, related to Figure 4. Our TUS results reported in the main text indicates that the effect of more reliable information in each condition was reduced following dmFPC TUS. To directly show this effect, we conducted another linear mixed effect model (see LMM4 in Methods) on incongruent trials separately for Face and Object conditions and compared each active stimulation condition versus Sham. In this model, the animals' choice was introduced as the dependent variables were (1) the direction of the reliable information in each condition (0 and 1 for left and right side), (2) ROI (0 for sham and 1 for any other active stimulation condition), and the interaction between these two regressors. It should be noted that this analysis is a confirmatory analysis and is not orthogonal to our accuracy analyses which we presented above and does not provide any information above and beyond suggested by the previous analysis but clarifies its interpretation. Consistent with our accuracy analysis, we found that in dmFPC TUS the effect of face was significantly lower than Sham in the incongruent trials of the Face condition, (β±95% CI=-.15±.09, $F(1,546)=14.3$ $p<.001$,HBC) and also than EVC (β±95% CI=-.11±.09, $F(1,547)=6.44$ $p=.01$,HBC). The effect of the face was also lower after mSTS TUS than Sham (β±95% CI=-.08±.06, $F(1,550)=7.38$ p<. 01). Similarly, in the incongruent trials of the Object condition, the face had a larger effect on choice after dmFPC TUS than Sham (β±95% CI=.04±.03, $F(1,584)=6.18$ $p=.01$,HBC) and also EVC compared to Sham (β±95% CI=-.03±.03, $F(1,579)=4.72$ $p=.03$, HBC). Finally, we repeated our LMM1 (the linear mixed effect model that we used to analyse our behavioural data of experiment 2) separately for each condition (Face or Object) and each TUS condition (Sham, dmFPC, mSTS, Sham). Consistent with the result of experiment 2, we found that in all conditions there was a significant impact of the more reliable information on choice (See Table).

Condition	Face	Object
Following correct trials	88±6(%)	94±5(%)
Following error trials	83 ± 19(%)	94±12(%)

Table S4: Investigating the effect of learning in experiment 2, related to Figure 2.

As we mentioned in our task description, the animals were tested once they reached near optimal performance. The animals' performance in the task (shown in Figure 2) supports this claim. Therefore, the computational problem that the animals encountered during the task was to weigh each source of information according to its reliability, but not learning the reliabilities. The reliability of each source of information was pre-learned during training. The weights could have been stored in terms of Q-values if we assume a reinforcement learning algorithm was recruited by the animals during training. It could be argued that the animals updated the weights during the task. However, if the animals updated the pre-learned weights during the task or were following a win-stay-lose-shift (WSLS) strategy, we would expect an effect of previous outcome on the current choice (effect of outcome on trials t-1 on the choice on trial t). In addition, if the animals were learning the task, we would expect their choice on the very first trial of each condition to be random. We tested both predictions. First, on the very first trial of the Face condition, the animals followed the face in 42 out of 46 sessions. The animals followed the better object in 42 out of 46 sessions in the first trial of the Object condition as well. These results indicate that the animals had learned the value/reliability of each source of information almost perfectly before doing the task, consistent with our training procedure and our claim in the manuscript. It should be noted that we used different pairs of objects and faces for reliable and unreliable objects and conditions, respectively.

We then went on to test whether there was any effect of previous outcome on choice or any evidence for WSLS strategy in the animals' behaviour. We first computed the proportion of trials on which animals chose the more reliable source of information on each condition (face direction in the Face condition and object direction in the Object condition) separately for trials on which the reliable source of information was correct or wrong on the previous trial. We argued that if the animals updated their value estimate after observing an outcome, we would expect the animals to choose the alternative information more often after wrong compared to correct trials. We found that in the Face condition in 83 ± 19(%) (mean ± standard deviation) of the trials the animals chose the face direction again after it had led to the wrong choice in the previous trial. Obviously, this is significantly above chance level ($w=893$, $p<.0001$). In the same condition, the animals followed the face direction after it led to the correct choice in the previous trial in 88±6(%) (m±std) of the trials. Comparing choosing face direction following correct and wrong trials showed no significant difference between the two trial types ($w=432$, $p=.32$).

We observed the same pattern in the Object condition: In 94±12(%) (m±std) of the trials the animals chose the same object that led to the wrong choice in the previous trial (test against chance level, $w=990$, $p<.0001$). The animals followed the same object after it led to the correct choice in the previous trial in 94±5(%) (m±std) of the trials. Again, with no difference in choosing the same object between the two trial types ($W=287$, $p=.76$).

These results indicate that there was no effect of previous outcome on current choice, consistent with the suggestion that while object and face values may initially have been

malleable when they were first encountered and during learning, this was no longer the case by the end of our training procedure. By this stage animals had learned the value/reliability of the face cues and the object cues which remained constant. Finally, we combined both conditions and conducted a regression model in which we predicted the animals' choices following the more reliable information on the current trial as a function of its accuracy on the previous trial. We conducted this model on the first 10 trials of each condition, where any potential learning is more likely to happen. Consistent with the analysis that we reported above, there was no effect of previous outcome on current choice (beta±95%CI= 0±.22, p=.93).

Supplementary references:

1. Van Essen, D.C., and Dierker, D.L. (2007). Surface-based and probabilistic atlases of primate cerebral cortex. Neuron *56*, 209–225.

2. Chau, B.K., Sallet, J., Papageorgiou, G.K., Noonan, M.P., Bell, A.H., Walton, M.E., and Rushworth, M.F. (2015). Contrasting roles for orbitofrontal cortex and amygdala in credit assignment and learning in macaques. Neuron *87*, 1106–1118.

3. Folloni, D., Fouragnan, E., Wittmann, M.K., Roumazeilles, L., Tankelevitch, L., Verhagen, L., Attali, D., Aubry, J.-F., Sallet, J., and Rushworth, M.F. (2021). Ultrasound modulation of macaque prefrontal cortex selectively alters credit assignment–related activity and behavior. Sci. Adv. *7*, eabg7700.

4. Rudebeck, P.H., Saunders, R.C., Lundgren, D.A., and Murray, E.A. (2017). Specialized representations of value in the orbital and ventrolateral prefrontal cortex: desirability versus availability of outcomes. Neuron *95*, 1208–1220.

5. Murray, E.A., and Rudebeck, P.H. (2018). Specializations for reward-guided decision-making in the primate ventral prefrontal cortex. Nat. Rev. Neurosci. *19*, 404–417.

6. Tsujimoto, S., Genovesio, A., and Wise, S.P. (2009). Monkey orbitofrontal cortex encodes response choices near feedback time. J. Neurosci. *29*, 2569–2574.

7. Miller, K.J., Botvinick, M.M., and Brody, C.D. (2022). Value representations in the rodent orbitofrontal cortex drive learning, not choice. Elife *11*, e64575.

8. Seltzer, B., and Pandya, D.N. (1989). Intrinsic connections and architectonics of the superior temporal sulcus in the rhesus monkey. J. Comp. Neurol. *290*, 451–471.

9. Yeterian, E.H., Pandya, D.N., Tomaiuolo, F., and Petrides, M. (2012). The cortical connectivity of the prefrontal cortex in the monkey brain. Cortex *48*, 58–81.

10. Gregoriou, G.G., Rossi, A.F., Ungerleider, L.G., and Desimone, R. (2014). Lesions of prefrontal cortex reduce attentional modulation of neuronal responses and synchrony in V4. Nat. Neurosci. *17*, 1003–1011.

11. Bichot, N.P., Xu, R., Ghadooshahy, A., Williams, M.L., and Desimone, R. (2019). The role of prefrontal cortex in the control of feature attention in area V4. Nat. Commun. *10*, 5727.

12. Xu, R., Bichot, N.P., Takahashi, A., and Desimone, R. (2022). The cortical connectome of primate lateral prefrontal cortex. Neuron *110*, 312–327.

13. Nguyen, M.N., Hori, E., Matsumoto, J., Tran, A.H., Ono, T., and Nishijo, H. (2013). Neuronal responses to face-like stimuli in the monkey pulvinar. Eur. J. Neurosci. *37*, 35–51.

14. Saalmann, Y.B., Pinsk, M.A., Wang, L., Li, X., and Kastner, S. (2012). The pulvinar regulates information transmission between cortical areas based on attention demands. science *337*, 753–756.

15. Romanski, L.M., Giguere, M., Bates, J.F., and Goldman-Rakic, P.S. (1997). Topographic organization of medial pulvinar connections with the prefrontal cortex in the rhesus monkey. J. Comp. Neurol. *379*, 313–332.

16. Boshra, R., and Kastner, S. (2022). Attention control in the primate brain. Curr. Opin. Neurobiol. *76*, 102605.
