# Supplementary material for: A frontopolar-temporal circuit determines the impact of social information in macaque decision making
Source: Neuron. 2024 Jan 3;112(1):84–92.e6. doi: 10.1016/j.neuron.2023.09.035 (PMC10914637; doi:10.1016/j.neuron.2023.09.035)
Supplement: Document S2. Article plus supplemental information [file mmc2.pdf]

Neuron

Report

A frontopolar-temporal circuit determines the impact of social information in macaque decision making

Highlights

- Macaques' dmFPC activity changes with the reliability of social information
- mSTS-dmFPC interaction occurs when social information influences behavior
- Disruption of dmFPC decreases the impact of social information when it is reliable
- Disruption of dmFPC increases the impact of social information when it is unreliable

Authors

Ali Mahmoodi, Caroline Harbison, Alessandro Bongioanni, ..., Jerome Sallet, Nima Khalighinejad, Matthew F.S. Rushworth

Correspondence

ali.mahmoodi1367@gmail.com

In brief

Mahmoodi et al. identify a neural circuit in macaques that comprises dorsomedial frontopolar cortex (dmFPC) and mid-superior temporal sulcus (mSTS) for regulating the impact of social information according to its reliability. Disruption of dmFPC and mSTS produces distinct changes in social information use.

Mahmoodi et al., 2024, Neuron *112*, 84–92
January 3, 2024 © 2023 The Author(s). Published by Elsevier Inc.
https://doi.org/10.1016/j.neuron.2023.09.035

CellPress

CellPress
OPEN ACCESS

Neuron

Report

A frontopolar-temporal circuit determines the impact of social information in macaque decision making

Ali Mahmoodi,[1,6,*] Caroline Harbison,[1] Alessandro Bongioanni,[1,2] Andrew Emberton,[3] Lea Roumazeilles,[1] Jerome Sallet,[1,4] Nima Khalighinejad,[1,5] and Matthew F.S. Rushworth[1,5]
[1]Wellcome Centre for Integrative Neuroimaging, Department of Experimental Psychology, University of Oxford, Oxford, UK
[2]Cognitive Neuroimaging Unit, CEA, INSERM, Université Paris-Saclay, NeuroSpin Center, 91191 Gif/Yvette, France
[3]Department of Biomedical Services, University of Oxford, Oxford, UK
[4]Univ Lyon, Université Lyon 1, Inserm, Stem Cell and Brain Research Institute, U1208 Bron, France
[5]These authors contributed equally
[6]Lead contact
*Correspondence: ali.mahmoodi1367@gmail.com
https://doi.org/10.1016/j.neuron.2023.09.035

SUMMARY

When choosing, primates are guided not only by personal experience of objects but also by social information such as others' attitudes toward the objects. Crucially, both sources of information—personal and socially derived—vary in reliability. To choose optimally, one must sometimes override choice guidance by personal experience and follow social cues instead, and sometimes one must do the opposite. The dorsomedial frontopolar cortex (dmFPC) tracks reliability of social information and determines whether it will be attended to guide behavior. To do this, dmFPC activity enters specific patterns of interaction with a region in the mid-superior temporal sulcus (mSTS). Reversible disruption of dmFPC activity with transcranial ultrasound stimulation (TUS) led macaques to fail to be guided by social information when it was reliable but to be more likely to use it when it was unreliable. By contrast, mSTS disruption uniformly downregulated the impact of social information on behavior.

INTRODUCTION

When faced with a choice, we often rely on several cues to guide our decision. In some cases, our behavior is directed by our own prior experience. For example, when choosing a meal at a restaurant, we may select a dish that we enjoyed previously. Sometimes, however, we may have reservations about the reliability or relevance of such experience. In this example, when traveling abroad, we instead place particular reliance on the opinions of others when choosing a dish. Human studies have demonstrated the impact of such social influence on behavior,[1–7] whereas similar patterns have been observed in other non-human primates. When vervet monkeys in the wild are offered two different foods that are identical in taste but different in color, they choose only the food color that they observe others eating.[8] Moreover, like the human diner abroad, vervets become more susceptible to social influence (i.e., opinion of others) when in unfamiliar territory.

Stimulus enhancement is believed to be the underlying mechanism of social influence in monkeys.[9] During stimulus enhancement, a monkey directs its behavior more toward a stimulus or object when it sees others doing the same.[9] Consistent with this account, neural activity in human and non-human primates reflects aspects of the actions of others,[2,10–13] but there is uncertainty about the impact that such activity has on the observer's behavior.[14] In humans, social information has a higher impact when it is more reliable or when the individuals are less certain about their own decisions.[7,15,16] Whether and how non-human primates shift flexibly from private to social information remain elusive. Here, we aim to address these questions, examining the way in which social influence may be accepted in some circumstances but not in others and its neural basis.

Another animal's face direction is an important cue in social situations.[17,18] Making eye contact with other animals and following their face direction can be used to infer what they might be about to do.[19–22] Monitoring where another animal's attention is directed may also be used to gain information about the environment that can be exploited by the observer. However, just like other sources of information, the reliability of social information varies. How the non-human primate's brain evaluates the reliability of social information to improve its decision making has not been studied. To search for brain mechanisms for evaluating and using social information, we trained macaques to perform a series of behavioral tasks in a magnetic resonance imaging (MRI) scanner while we recorded activity across the whole brain. We then used minimally invasive transcranial ultrasound stimulation

 © 2023 The Author(s). Published by Elsevier Inc.
This is an open access article under the CC BY license (http://creativecommons.org/licenses/by/4.0/).

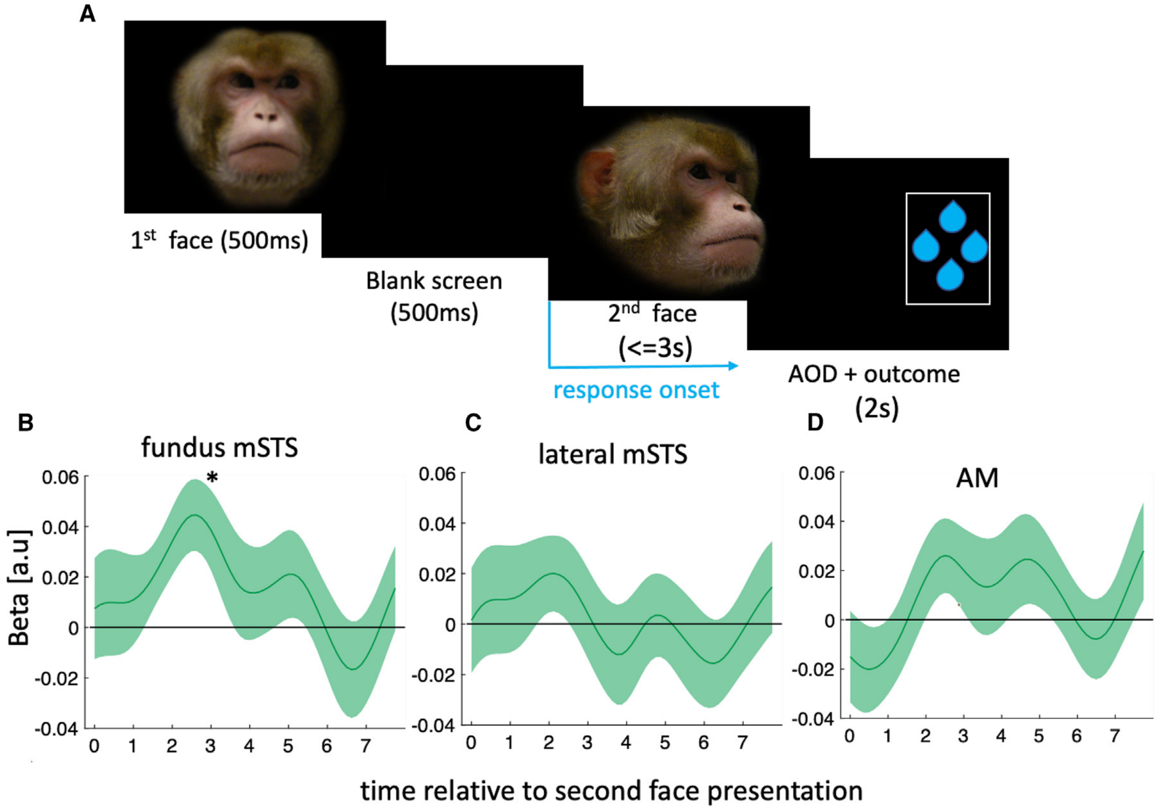

Figure 1. Experiment 1: face direction is encoded in mSTS
(A) Two faces appeared consecutively (separated by 500 ms blank screen delay). Animals had 3 s from onset of the second face to choose which side it was facing.
(B–D) Response of mSTS fundus (B), lateral mSTS (C), and AM (D) to identical versus different face direction. y axis indicates β_1 in Equation 7. In (B)–(D), the line and the shaded area represent mean and standard error of the mean (SEM), respectively.*$p_{FDR} < 0.05$.

(TUS) to examine the impact of transient neural disruption on behavior.

RESULTS

Experiment 1

Before examining how social influence is exerted in decision making, or is upregulated or downregulated, we sought to identify brain areas encoding other animals' face direction. On each trial, a monkey's face appeared at the screen center for 500 ms (first face), followed by 500 ms of blank screen. The face was either *neutral* (gazing forward) or *directed* (gazing left or right). Afterward, a second face, always directed, appeared. Animals chose the side the second face was gazing at (Figure 1A). The direction of the second face could have been similar or dissimilar to the first face. The experimental design was optimized to exploit repetition suppression (a reduction in neural activity due to repeated presentation of a stimulus feature) to find brain areas encoding face direction by looking for areas in which activity was reduced when the second face direction was the same as, as opposed to different to, the first face. We primarily focused on three regions of interest (ROIs) in or adjacent to the superior temporal sulcus (STS) known to contain face-responsive neurons. The first two ROIs were in the fundus of the mid-portion of the STS (mSTS, Figure S1 for coordinates) and a laterally adjacent region where STS meets the inferior temporal gyrus (coordinates taken from an independent study[23]). A third region was close to the anterior medial (AM) face patch.[24,25]

In all time-course analyses, we applied Holm-Bonferroni correction (HBC) for multiple comparison across ROIs and false discovery rate (FDR) across time points. Examination of ROI activity using repetition suppression (Figure S1 legend) indicated that at the time of the second face, only mSTS fundus activity exhibited significant face direction repetition suppression (Figure 1B, $p_{FDR} = 0.03$, HBC), whereas neither the more lateral mSTS ($p_{FDR} > 0.05$) nor AM ($p_{FDR} > 0.05$) reached significance (Figures 1C and 1D). The results suggest that mSTS fundus (henceforth mSTS) encodes face direction (Figure S1 summarizes whole-brain results).

Experiment 2

Next, we examined how animals switch between reliance and non-reliance on social information during decision making (Figures 2A and 2B). Each monkey (n = 3) performed 16 functional MRI (fMRI) sessions. At the beginning of each trial, an image of a monkey's face appeared in the center of the screen

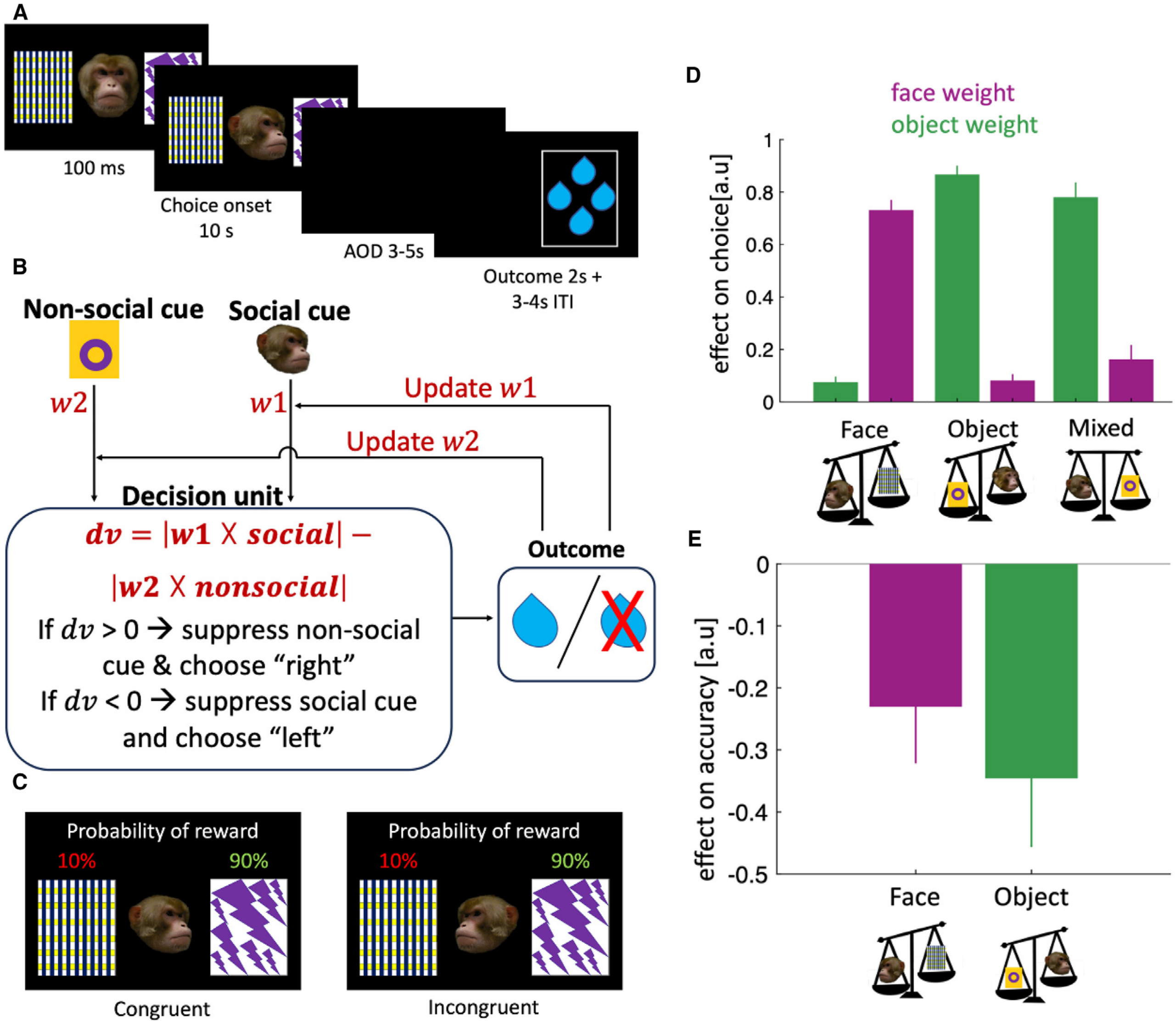

Figure 2. Experiment 2: task design and behavioral results

(A) Animals were presented with two objects on either side of the screen and a face gazing at one of the two objects.

(B) The main computational problems in social situations can be divided into learning stage and decision stage. In the learning stage, the reliability of social information should be learned (using any learning algorithm). In the decision stage, the learned reliability should be used to regulate the weight of the social information. In this study, the computational problem that the animals encountered during the task was to solve the decision stage as the animals were tested once they reached near optimal performance. Therefore, their task was to weigh each source of information according to its reliability and choose the more reliable information.

(C) In all conditions, a trial was marked as incongruent if the face was directed away from the better of the two objects. In these trials, it was critical to weigh each source of information according to its reliability.

(D) Beta coefficients from linear mixed-effect models are plotted for different experimental conditions (β_2 and β_3 in Equation 1).

(E) Beta coefficients indicating the impact of incongruency on accuracy (β_2 in Equation 2).

In (D) and (E) error bars indicate 95% confidence interval.

gazing forward, with an object on either side of the face (Figure 2A). After 100 ms, the face turned to one object, and animals chose the side they thought would lead to reward. The experiment was divided into three conditions/blocks, each containing 50 trials. In the face condition, face reliability was high (the face gazed at the rewarded object in 90% of trials), whereas the better and the worse objects each had low reliabilities. In the object condition, object reliability was high (90%), whereas face reliability was low. In the mixed condition, both face and object reliabilities were high. Prior training with both face and object stimuli ensured that animals' decisions reflected the reward probabilities of the different pairs of objects and faces (STAR Methods). We used different pairs of objects and faces for the reliable and unreliable conditions (i.e., one pair of objects for

CellPress
OPEN ACCESS

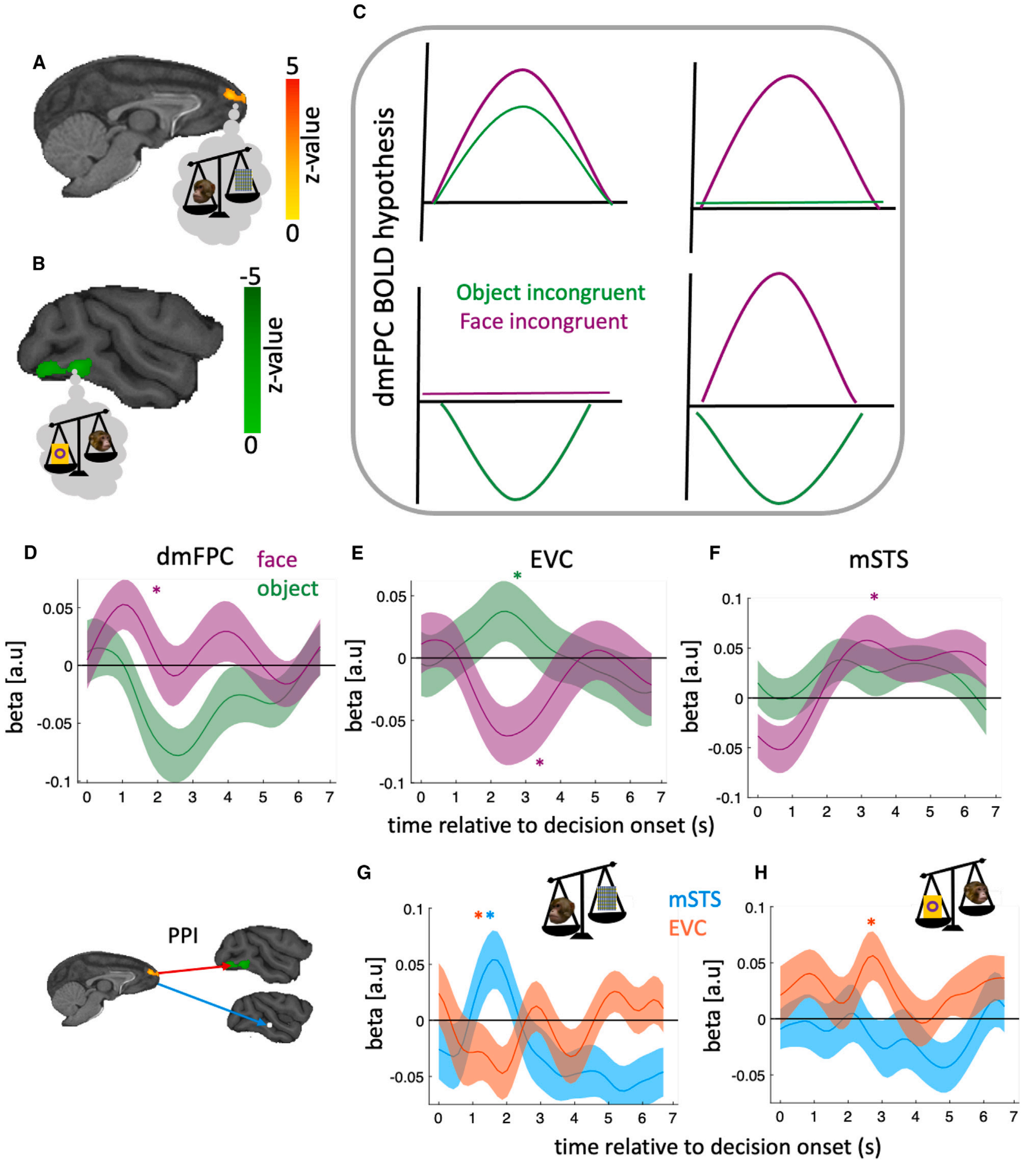

Figure 3. dmFPC involvement in our task

(A and B) Whole-brain analysis showed that dmFPC peak Montreal Neurological Institute (MNI) coordinate [8 27 14] (A) and EVC peak MNI coordinate [2 −43 1] (B) were the only areas in which activity was significantly different between the face and the object conditions on incongruent trials. The activation maps were obtained by subtracting the incongruent trials in the face condition from the incongruent trials in the object condition.

(C) The positive effect in dmFPC could be due to different possible patterns of activity. Top left, if dmFPC is positively correlated with incongruency in both conditions, we expect positive dmFPC response to incongruency in both conditions, but more so in the face condition. Such a pattern of activity would be consistent with dmFPC encoding incongruency. Top-right, if dmFPC was only critical in incongruent face trials, we expect positive correlation with incongruency

(legend continued on next page)

the object and mixed conditions and a different pair for the face condition, and one face for the face and mixed conditions and another for the object condition). Thus, macaques could infer the current condition by the object and face identities and their associated reliabilities. A distinct feature of our task design is that in some trials (regardless of the condition), the face was directed away from the better of the two objects. We labeled these trials as "incongruent" trials, as opposed to "congruent" trials where the face was directed toward the better of the two objects (Figure 2C). There was no conflict between the two sources of information in the congruent trials, whereas in the incongruent trials, there was conflict. The animals should have decided which source of information to follow according to the reliability of each source.

We used mixed-effects logistic regression to quantify the impact of faces and objects on choices in each condition (linear mixed-effect model [LMM1] in STAR Methods). In the face condition, choice was predominantly influenced by face direction (Figure 2D, $\beta \pm$ 95% confidence interval [CI] = 0.73 ± 0.04, $F(1, 45) = 1{,}229$, $p < 0.001$); however, interestingly, there was also a significant effect of object (Figure 2D, $\beta \pm$ 95% CI = 0.07 ± 0.03, $F(1, 43) = 26$, $p < 0.001$). This pattern was reversed in the object condition: choices were predominantly influenced by objects (Figure 2D, $\beta \pm$ 95% CI = 0.86 ± 0.03, $F(1, 46) = 2{,}134$, $p < 0.001$), but there was also a small but significant effect of face ($\beta \pm$ 95% CI = 0.08 ± 0.03, $F(1, 52) = 37$, $p < 0.001$). The choice pattern in the mixed condition resembled that seen in the object condition; choices were largely determined by objects (Figure 2D, $\beta \pm$ 95% CI = 0.78 ± 0.07, $F(1, 46) = 569$, $p < 0.001$), but there was also a significant effect of face ($\beta \pm$ 95% CI = 0.16 ± 0.06, $F(1, 46) = 33$, $p < 0.001$). The greater reliance placed on objects in the mixed condition, even when face and object reliabilities were equivalent, implies that choosing based on object information was the animals' default strategy, and they only deviated from it when object information was less reliable than face information. The effect of face in the object condition and the effect of object in the face condition imply that accuracy should have decreased in incongruent trials (Figure 2C, trials in which the face was not gazing at the more reliable object; LMM2 in STAR Methods). Consistent with our prediction, in both conditions, incongruency impaired accuracy (Figure 2E, face condition: $\beta \pm$ 95% CI = −0.23 ± 0.09, $F(1, 45) = 24$, $p < 0.001$, object condition: $\beta \pm$ 95% CI = −0.34 ± 0.11, $F(1, 53) = 37$, $p < 0.001$).

We then turned to the fMRI data to understand how the brain represents and weighs up social and non-social information and how it facilitates (suppresses) the impact of the more (less) reliable information on choice. Notably, only in incongruent trials can we unambiguously infer what information (social or non-social) guided choice. Using a whole-brain general linear model (GLM) (GLM3 in STAR Methods), we divided our trials into three categories as follows: incongruent trials in the face condition when animals followed face direction, incongruent trials in the object condition when animal chose against the information provided by the face, and all congruent trials.

Activity in extrastriate visual cortex (EVC), including areas V4 and TEO (henceforth EVC), was significantly higher when monkeys were focusing on the visual objects themselves rather than on the oriented monkey face; it was more active in the incongruent object versus incongruent face condition (Figure 3B). This latter finding is consistent with previous demonstrations that this region is important when monkeys learn about and attend to visual objects.[26–29] By contrast, however, we found that dorsomedial frontopolar cortex (dmFPC) was the only area that had significantly higher activity in the incongruent face versus incongruent object condition (Figure 3A; Table S1 for details). Notably, the difference in dmFPC activity between face and object conditions was specific to incongruent trials: there was no difference in dmFPC activity between the two conditions in congruent trials (Figure S2A).

The positive effect in dmFPC could arise because of distinct patterns of activity (Figures 3C–3F). To tease apart these possibilities, we performed additional time-course analyses of dmFPC activity (see STAR Methods). Since we had very few incongruent trials in the mixed condition, we restricted our analyses to face and object conditions. We ran separate regression models for face and object conditions. In these models, congruency (1 and 0 for incongruent and congruent trials, respectively) and reaction time were included as independent variables, and blood-oxygen-level-dependent (BOLD) signal as a dependent variable. We found that dmFPC activity was more positive in the incongruent face condition when the face was followed (Figure 3G, $p_{FDR} = 0.01$, HBC), whereas it was more negative in the incongruent object condition when the face was not followed (Figure 3G, $p_{FDR} = 0.01$, HBC). An approximately opposite effect was observed in EVC: it was negative in the incongruent face condition (Figure 3H, $p_{FDR} = 0.02$, HBC), but not significantly different from zero in the incongruent object trials (Figure 3H, $p_{FDR} > 0.05$, HBC). Finally, mSTS, the area that encoded face direction in experiment 1, was more active in the incongruent face condition (Figure 3I, $p_{FDR} = 0.01$, HBC) but not in the incongruent

in the face condition, but no significant relationship with incongruency in the object condition. This would suggest that dmFPC facilitates the impact of social information when it is reliable. Bottom-left, if dmFPC was only critical in incongruent object trials, we expect a negative correlation between dmFPC activity and incongruency in the object condition, but no significant relationship with incongruency in the face condition. This would suggest dmFPC suppresses the impact of object information when it is unreliable. Bottom-right, if dmFPC relationship with incongruency was different between the two conditions, we expect positive correlation with incongruency in the face condition and negative correlation in the object condition. This would suggest that dmFPC regulates the impact of social information according to its reliability—promoting social information in the face condition when it is reliable and suppressing the object information, which is attended by default, when it is unreliable. In addition, it would suggest that dmFPC suppresses social information when it is unreliable in the object condition.
(D) In incongruent face trials, dmFPC activity increased (compared with congruent trials), whereas it decreased in incongruent object trials.
(E) The opposite pattern was observed in EVC.
(F) mSTS activity as a function of incongruency in each condition.
(G) Functional connectivity between dmFPC and face and object related areas in each condition.
(H) Same as (F) but in the object condition. In (D)–(F), y axis indicates β_1 in Equation 8. In (G)–(H), y axis indicates β_3 in Equation 9.
In (D)–(H), the line and the shaded area represent mean and standard error of the mean (SEM), respectively. *$p_{FDR} < 0.05$.

CellPress
OPEN ACCESS

object condition (Figure 3I, $p_{FDR} > 0.05$, HBC), with no difference between the two conditions ($p_{FDR} > 0.05$).

Next, we examined whether functional connectivity between dmFPC, on the one hand, and mSTS or EVC, on the other hand, varied as a function of experimental condition (face or object, see the Figure S2 legend for details). Here, we predicted dmFPC activity using incongruency as the psychological variable and mSTS activity as the physiological variable. Consistent with our prediction, in the face condition, dmFPC connectivity with mSTS increased in incongruent trials (Figure 3J, p_{FDR} = 0.008, HBC), whereas its connectivity with EVC did not change (Figure 3J, $p_{FDR} > 0.05$). In contrast, in the object condition, dmFPC-EVC connectivity increased in incongruent trials (Figure 3K, p_{FDR} = 0.04, HBC), whereas FPC-mSTS connectivity was not different from zero (Figure 3K, $p_{FDR} > 0.05$).

So far, our findings suggest that dmFPC facilitates the impact of social information on choice when it is reliable but limits its impact when it is unreliable, an interpretation that we return to in experiment 3, below. Notably, the "reliability assessment" role proposed for dmFPC contrasts with top-down control over sensory processing in the absence of ambiguity and a need to infer the reliability of an information source. Contrary to what may have been expected, ventral prearcuate cortex, an area associated with exerting top-down control over other sensory areas played little role in our task.[30,31]

We performed additional analyses that were time-locked to outcome rather than decision time to test whether dmFPC activity patterns were specific to the process of decision making. We found that all dmFPC patterns of activity were specific to decision time and were not observable at outcome time (Figure S2). We repeated these analyses on another area of the prefrontal cortex (PFC), 47/12o. Unlike the dmFPC activity pattern found in our task, which is specific to decision time, 47/12o is important for learning and outcome processing.[32–34] In Figure S2, we show that 47/12o's activity pattern is not specifically related to decision. Therefore, dmFPC activity is most likely related to decision making itself or computational processes on which decision making depends (Figure 2B).

Experiment 3

Finally, we examined whether dmFPC was causally responsible for determining whether social information would guide decision making. The same three animals completed 12 more sessions consisting of face and object conditions (50 trials each) after receiving offline TUS or sham. In the active sessions, TUS was applied before behavioral testing to one of the three areas (dmFPC, mSTS, or EVC). In sham sessions, no stimulation was applied. Each animal completed four rounds of data collection in each of the four experimental conditions (STAR Methods).

Different hypotheses about the role of dmFPC predict different deficits in behavior (Figures 4A–4C). We ran LMMs (LMM3 in STAR Methods), in which we directly compared each stimulation site with sham. The models were run separately for congruent and incongruent trials. We found that in the incongruent face condition, there was a significant reduction in accuracy with dmFPC-TUS compared with sham (Figure 4D; $\beta \pm 95\%$ CI = -0.05 ± 0.03, $F(1, 548) = 11$, $p < 0.001$, HBC). We found a similar reduction in accuracy when comparing mSTS-TUS versus sham (Figure 4D; $\beta \pm 95\%$ CI = -0.03 ± 0.03, $F(1, 552) = 4.68$, $p = 0.03$, HBC), whereas there was no effect of EVC-TUS on accuracy (Figure 4D; $\beta \pm 95\%$ CI = -0.01 ± 0.02, $F(1, 551) = 1$, $p = 0.31$).

Critically, stimulation in no area affected accuracy in congruent trials (Table S2). We directly compared TUS effects on accuracy between congruent and incongruent trials. As incongruency has a negative effect on accuracy (Figure 2E), we compared reduction in accuracy in incongruent trials across areas. If TUS specifically affects incongruent trials, the negative effect of incongruency should be more pronounced during TUS compared with sham. We therefore added incongruency to our linear regression model and compared dmFPC-TUS versus sham. We found a significant negative interaction between TUS and incongruency ($\beta \pm 95\%$ CI = -0.02 ± 0.02, $F(1, 1{,}179) = 5$, $p = 0.02$) meaning that compared with sham, dmFPC-TUS had a more negative impact on accuracy in incongruent than in congruent trials. This difference did not reach significance in the mSTS-TUS versus sham comparison ($\beta \pm 95\%$ CI = -0.01 ± 0.02, $F(1, 1{,}176) = 1.67$, $p = 0.19$).

We tested our hypotheses in the object condition where we expected a reduction in accuracy following dmFPC and EVC-TUS. We found a significant reduction in accuracy in dmFPC versus sham (Figure 4E, $\beta \pm 95\%$ CI = -0.06 ± 0.06, $F(1, 584) = 5.5$, $p = 0.03$, HBC) and in EVC versus sham (Figure 4E, $\beta \pm 95\%$ CI = -0.06 ± 0.06, $F(1, 584) = 4.7$, $p = 0.03$, HBC), whereas, as expected, there was no effect in mSTS versus sham (Figure 4E, $\beta \pm 95\%$ CI = -0.01 ± 0.07, $F(1, 579) = 0.23$, $p = 0.62$, HBC). We found that these effects were not significantly different between congruent and incongruent trials in the object condition. As before, we quantified this by asking whether reduction in accuracy in incongruent trials was more pronounced in dmFPC versus sham ($\beta \pm 95\%$ CI = -0.01 ± 0.02, $F(1, 1{,}182) = 0.77$, $p = 0.15$) and EVC versus sham ($\beta \pm 95\%$ CI = -0.01 ± 0.02, $F(1, 1{,}161) = 3.12$, $p = 0.07$). However, after combining face and object conditions, we found that the reduction in accuracy was significantly higher in incongruent compared with congruent trials following dmFPC-TUS ($\beta \pm 95\%$ CI = -0.02 ± 0.01, $F(1, 2{,}392) = 7.04$, $p = 0.008$). On repeating LMM3 for congruent trials, we found no effect of TUS on accuracy (Table S2). Therefore, dmFPC-TUS specifically targeted incongruent, but not congruent, trials. Finally, we show that the impairment in the incongruent trials indicates that the effect of more reliable information in each condition was reduced following TUS (Table S3).

DISCUSSION

dmFPC evaluates and determines the impact of social information on choice by adjusting its activity according to the reliability of the social information. Its activity was responsive to a combination of congruency and reliability: it was highest when social information was reliable and incongruent to the non-social information and at its nadir when social information was unreliable and incongruent to the non-social information. Consistent with these findings, our TUS results showed that dmFPC-TUS led macaques to fail to be guided by social information when it was reliable but to be more likely to use

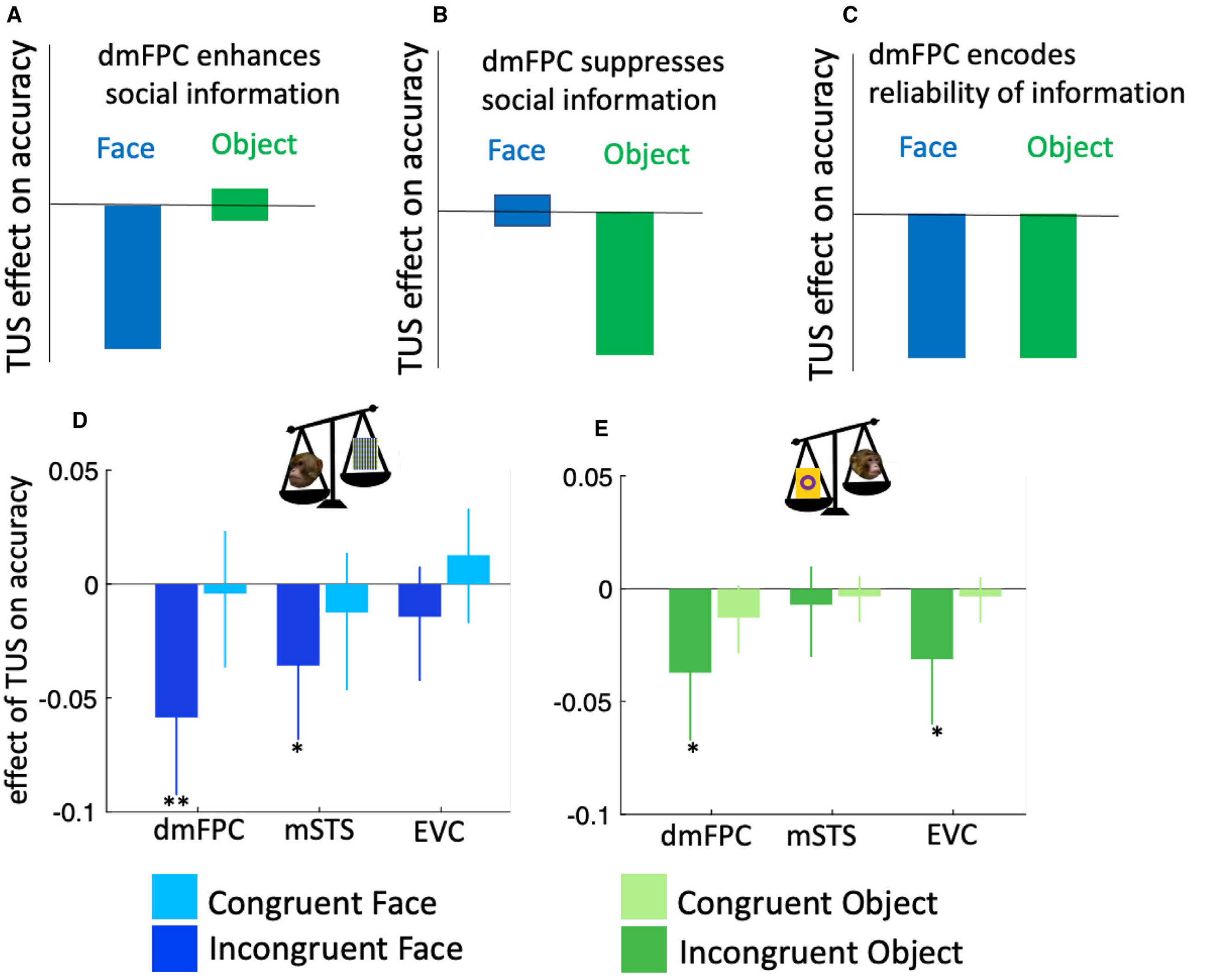

Figure 4. Result of TUS experiment (experiment 3) on different regions

(A) If dmFPC only enhances social information, its disruption would impair performance in the face, but not in the object, condition.

(B) If dmFPC is only important in suppressing social information, its disruption would impair performance only in the object condition.

(C) If dmFPC regulates the impact of social information based on its reliability, its disruption should impair performance in both face and object conditions.

(D and E) We used linear regression models to quantify the impact of TUS on animals' accuracy in face (D) and object (E) conditions, separately for congruent and incongruent trials. The y axis indicates β_2 in Equation 3, which captures difference in accuracy in each stimulation condition against sham.

In (D) and (E) error bars indicate 95% confidence interval. * p <. 05, ** p <. 001

it when it was unreliable. Temporoparietal junction (TPJ) and dorsomedial PFC (dmPFC)—the human homologs of mSTS and dmFPC, respectively—are prominent in many social tasks.[5,35–37] It has been suggested that dmPFC in humans may be important for inferring others' mental state.[38,39] Others have suggested a more general role for this area in social cognition,[40] whereas a recent study has suggested that dmPFC is involved in separating the currently relevant social information from the currently irrelevant ones.[37] Our study shows that dmFPC in monkeys might play a similar role: separating reliable information from the unreliable. According to this view, dmFPC plays a general role in weighing information according to its relevance, regardless of whether the relevance is imposed by context. Such a function is broadly consistent with dmFPC's tracking of the significance of alternative goals and facilitation of behavior switching.[41,42] This can be tested in future studies using more general cue-combination tasks[43] to develop computational theories about the broader role of frontal areas like dmFPC. Finally, it is likely that dmFPC is involved not only in weighing social information according to reliability but also in learning the reliability itself. However, our study was not designed to answer this question; our subjects performed the task inside the scanner after having already learned about the reliabilities of the different stimuli and, consequently, reached near optimal performance during pre-task training (Table S4).

CellPress
OPEN ACCESS

It has been shown that the brain computes a weighted average (according to reliability) to combine different sources of information.[16,43] Our study indicated that the information provided by sensory areas (such as mSTS and EVC) is processed and moderated by frontal areas. We found that dmFPC activity was modulated by a combination of the reliability and congruency of the social information: it was at its peak when the social information was reliable and incongruent to the non-social information, and its nadir when social information was unreliable and incongruent to the non-social information. Critically, this process of reliability-based weighting of information was achieved through the interaction between frontal and temporal-cortical areas. Our study provides a computational account for how such a reliability-based weighting of information is implemented in the brain, which might extend to non-social contexts as well.

Previous studies have shown that certain regions of the human and non-human primate brain encode and predict the actions of other individuals in their environment.[23,44] The primary use of this predictive mechanism has been linked to theory of mind. Our study, however, suggests that such information regarding others can also be used to guide the observing animals' own behaviors when it is more reliable than other information sources.

STAR★METHODS

Detailed methods are provided in the online version of this paper and include the following:

- KEY RESOURCES TABLE
- RESOURCE AVAILABILITY
  - Lead contact
  - Materials availability
  - Ethical compliance
  - Data and code availability
- METHOD DETAILS
  - Behavioral training
  - Experiment 1: encoding of face direction and identity
  - Experiment 2: impact of social information on choice
  - Experiment 3: causal manipulation using TUS
  - fMRI data acquisition and processing
  - TUS protocol
- QUANTIFICATION AND STATISTICAL ANALYSIS
  - Behavioral data analysis
  - fMRI data analysis
  - GLMs 1 & 2 (whole brain analysis for experiment 1)
  - GLM 3 (whole brain analysis for experiment 2)
  - Activity time course analysis
  - Creating Region of Interests (ROIs) for the time course analysis

SUPPLEMENTAL INFORMATION

Supplemental information can be found online at https://doi.org/10.1016/j.neuron.2023.09.035.

ACKNOWLEDGMENTS

The authors would like to thank Fateme Daneshzade for editing the face stimuli. N.K. is funded by BBSRC Discovery Fellowship (BB/W008947/1). M.F.S.R. is funded by BBSRC grant BB/W003392/1, Wellcome Trust grants 221794/Z/20/Z and 203139/Z/16/Z, and MRC grant MR/P024955/1.

AUTHOR CONTRIBUTIONS

A.M. and M.F.S.R. conceived the study. A.M., A.B., and M.F.S.R. designed experiment 1. A.M., J.S., N.K., and M.F.S.R. designed experiment 2. A.M., N.K., and M.F.S.R. designed experiment 3. A.M., C.H., A.E., L.R., and N.K. collected the data. J.S., N.K., and M.F.S.R. mentored animal training and testing. A.B. and N.K. prepared the TUS setup. A.M. analyzed behavioral and neural data with help from C.H., A.B., and N.K. A.M. wrote the first draft of the manuscript with input from C.H. and M.F.S.R. C.H., A.B., J.S., N.K., and M.F.S.R. contributed to the final version of the manuscript. All authors approved the final manuscript.

DECLARATION OF INTERESTS

The authors declare no competing interests.

Received: May 1, 2023
Revised: July 6, 2023
Accepted: September 26, 2023
Published: October 19, 2023

REFERENCES

1. Behrens, T.E., Hunt, L.T., Woolrich, M.W., and Rushworth, M.F. (2008). Associative learning of social value. Nature *456*, 245–249.
2. Park, S.A., Goïame, S., O'Connor, D.A., and Dreher, J.-C. (2017). Integration of individual and social information for decision making in groups of different sizes. PLoS Biol. *15*, e2001958.
3. Qi, Y., Liu, Z., Cao, S., Han, Y., Wang, Q., Liu, X., and Wu, H. (2023). Social value orientation modulates behavioral and neural responses to social influence. Hum. Brain Mapp. *44*, 3222–3231.
4. Chen, X., Liu, J., Luo, Y.-J., and Feng, C. (2023). Brain systems underlying fundamental motivations of human social conformity. Neurosci. Bull. *39*, 328–342.
5. Mahmoodi, A., Nili, H., Bang, D., Mehring, C., and Bahrami, B. (2022). Distinct neurocomputational mechanisms support informational and socially normative conformity. PLoS Biol. *20*, e3001565.
6. Mahmoodi, A., Bahrami, B., and Mehring, C. (2018). Reciprocity of social influence. Nat. Commun. *9*, 2474.
7. Mahmoodi, A., Bang, D., Olsen, K., Zhao, Y.A., Shi, Z., Broberg, K., Safavi, S., Han, S., Nili Ahmadabadi, M.N., Frith, C.D., et al. (2015). Equality bias impairs collective decision making across cultures. Proc. Natl. Acad. Sci. USA *112*, 3835–3840.
8. Van de Waal, E., Borgeaud, C., and Whiten, A. (2013). Potent social learning and conformity shape a wild primate's foraging decisions. Science *340*, 483–485.
9. Heyes, C.M. (1994). Social learning in animals: categories and mechanisms. Biol. Rev. Camb. Philos. Soc. *69*, 207–231. https://doi.org/10.1111/j.1469-185x.1994.tb01506.x.
10. Klucharev, V., Hytönen, K., Rijpkema, M., Smidts, A., and Fernández, G. (2009). Reinforcement learning signal predicts social conformity. Neuron *61*, 140–151.
11. Izuma, K., and Adolphs, R. (2013). Social manipulation of preference in the human brain. Neuron *78*, 563–573.
12. Báez-Mendoza, R., Mastrobattista, E.P., Wang, A.J., and Williams, Z.M. (2021). Social agent identity cells in the prefrontal cortex of interacting groups of primates. Science *374*, eabb4149.
13. Jamali, M., Grannan, B.L., Fedorenko, E., Saxe, R., Báez-Mendoza, R., and Williams, Z.M. (2021). Single-neuronal predictions of others' beliefs in humans. Nature *591*, 610–614.
14. Rizzolatti, G., and Sinigaglia, C. (2016). The mirror mechanism: a basic principle of brain function. Nat. Rev. Neurosci. *17*, 757–765.

15. Pescetelli, N., and Yeung, N. (2021). The role of decision confidence in advice-taking and trust formation. J. Exp. Psychol. Gen. *150*, 507–526.
16. Bahrami, B., Olsen, K., Latham, P.E., Roepstorff, A., Rees, G., and Frith, C.D. (2010). Optimally interacting minds. Science *329*, 1081–1085.
17. Kendon, A. (1967). Some functions of gaze-direction in social interaction. Acta Psychol. *26*, 22–63.
18. Emery, N.J. (2000). The eyes have it: the neuroethology, function and evolution of social gaze. Neurosci. Biobehav. Rev. *24*, 581–604.
19. Ong, W.S., Madlon-Kay, S., and Platt, M.L. (2021). Neuronal correlates of strategic cooperation in monkeys. Nat. Neurosci. *24*, 116–128.
20. Dal Monte, O., Chu, C.C.J., Fagan, N.A., and Chang, S.W.C. (2020). Specialized medial prefrontal–amygdala coordination in other-regarding decision preference. Nat. Neurosci. *23*, 565–574.
21. Dal Monte, O., Fan, S., Fagan, N.A., Chu, C.-C.J., Zhou, M.B., Putnam, P.T., Nair, A.R., and Chang, S.W.C. (2022). Widespread implementations of interactive social gaze neurons in the primate prefrontal-amygdala networks. Neuron *110*, 2183–2197.e7.
22. Gangopadhyay, P., Chawla, M., Dal Monte, O., and Chang, S.W.C. (2021). Prefrontal–amygdala circuits in social decision making. Nat. Neurosci. *24*, 5–18.
23. Roumazeilles, L., Schurz, M., Lojkiewiez, M., Verhagen, L., Schüffelgen, U., Marche, K., Mahmoodi, A., Emberton, A., Simpson, K., and Joly, O. (2021). Social prediction modulates activity of macaque superior temporal cortex. Sci. Adv. *7*, eabh2392.
24. Freiwald, W.A., and Tsao, D.Y. (2010). Functional compartmentalization and viewpoint generalization within the macaque face-processing system. Science *330*, 845–851.
25. Chang, L., and Tsao, D.Y. (2017). The code for facial identity in the primate brain. Cell *169*, 1013–1028.e14.
26. Kikuchi, R., and Iwai, E. (1980). The locus of the posterior subdivision of the inferotemporal visual learning area in the monkey. Brain Res. *198*, 347–360.
27. De Weerd, P., Peralta, M.R., Desimone, R., and Ungerleider, L.G. (1999). Loss of attentional stimulus selection after extrastriate cortical lesions in macaques. Nat. Neurosci. *2*, 753–758.
28. Buffalo, E.A., Bertini, G., Ungerleider, L.G., and Desimone, R. (2005). Impaired filtering of distracter stimuli by TE neurons following V4 and TEO lesions in macaques. Cereb. Cortex *15*, 141–151.
29. Bertini, G., Buffalo, E.A., De Weerd, P., Desimone, R., and Ungerleider, L.G. (2004). Visual responses to targets and distracters by inferior temporal neurons after lesions of extrastriate areas V4 and TEO. NeuroReport *15*, 1611–1615.
30. Wardak, C., Vanduffel, W., and Orban, G.A. (2010). Searching for a salient target involves frontal regions. Cereb. Cortex *20*, 2464–2477.
31. Bichot, N.P., Xu, R., Ghadooshahy, A., Williams, M.L., and Desimone, R. (2019). The role of prefrontal cortex in the control of feature attention in area V4. Nat. Commun. *10*, 5727.
32. Chau, B.K., Sallet, J., Papageorgiou, G.K., Noonan, M.P., Bell, A.H., Walton, M.E., and Rushworth, M.F. (2015). Contrasting roles for orbitofrontal cortex and amygdala in credit assignment and learning in macaques. Neuron *87*, 1106–1118.
33. Miller, K.J., Botvinick, M.M., and Brody, C.D. (2022). Value representations in the rodent orbitofrontal cortex drive learning, not choice. eLife *11*, e64575.
34. Rudebeck, P.H., Saunders, R.C., Lundgren, D.A., and Murray, E.A. (2017). Specialized representations of value in the orbital and ventrolateral prefrontal cortex: desirability versus availability of outcomes. Neuron *95*, 1208–1220.e5.
35. Sallet, J., Mars, R.B., Noonan, M.P., Neubert, F.-X., Jbabdi, S., O'Reilly, J.X., Filippini, N., Thomas, A.G., and Rushworth, M.F. (2013). The organization of dorsal frontal cortex in humans and macaques. J. Neurosci. *33*, 12255–12274.
36. Mars, R.B., Sallet, J., Neubert, F.-X., and Rushworth, M.F. (2013). Connectivity profiles reveal the relationship between brain areas for social cognition in human and monkey temporoparietal cortex. Proc. Natl. Acad. Sci. USA *110*, 10806–10811.
37. Mahmoodi, A., Nili, H., Harbison, C., Hamilton, S., Trudel, N., Bang, D., and Rushworth, M.F.S. (2023). Causal role of a neural system for separating and selecting multidimensional social cognitive information. Neuron *111*, 1152–1164.e6.
38. Van Overwalle, F. (2009). Social cognition and the brain: a meta-analysis. Hum. Brain Mapp. *30*, 829–858.
39. Yoshida, W., Dolan, R.J., and Friston, K.J. (2008). Game theory of mind. PLoS Comput. Biol. *4*, e1000254.
40. Saxe, R., and Powell, L.J. (2006). It's the thought that counts: specific brain regions for one component of theory of mind. Psychol. Sci. *17*, 692–699.
41. Mansouri, F.A., Buckley, M.J., Mahboubi, M., and Tanaka, K. (2015). Behavioral consequences of selective damage to frontal pole and posterior cingulate cortices. Proc. Natl. Acad. Sci. USA *112*, E3940–E3949. https://doi.org/10.1073/pnas.1422629112.
42. Mansouri, F.A., Koechlin, E., Rosa, M.G.P., and Buckley, M.J. (2017). Managing competing goals—a key role for the frontopolar cortex. Nat. Rev. Neurosci. *18*, 645–657.
43. Ernst, M.O., and Banks, M.S. (2002). Humans integrate visual and haptic information in a statistically optimal fashion. Nature *415*, 429–433.
44. Ninomiya, T., Noritake, A., and Isoda, M. (2021). Live agent preference and social action monitoring in the macaque mid-superior temporal sulcus region. Proc. Natl. Acad. Sci. USA *118*, e2109653118.
45. Tustison, N.J., and Avants, B.B. (2013). Explicit B-spline regularization in diffeomorphic image registration. Front. Neuroinform. *7*, 39.
46. Brainard, D.H. (1997). The psychophysics toolbox. Spat. Vis. *10*, 433–436.
47. Grill-Spector, K., and Malach, R. (2001). fMR-adaptation: a tool for studying the functional properties of human cortical neurons. Acta Psychol. *107*, 293–321.
48. Henson, R.N.A., and Rugg, M.D. (2003). Neural response suppression, haemodynamic repetition effects, and behavioral priming. Neuropsychologia *41*, 263–270.
49. Shahdloo, M., Schüffelgen, U., Papp, D., Miller, K.L., and Chiew, M. (2022). Model-based dynamic off-resonance correction for improved accelerated fMRI in awake behaving nonhuman primates. Magn. Reson. Med. *87*, 2922–2932.
50. Jenkinson, M., Beckmann, C.F., Behrens, T.E., Woolrich, M.W., and Smith, S.M. (2012). Fsl. Neuroimage *62*, 782–790.
51. Verhagen, L., Gallea, C., Folloni, D., Constans, C., Jensen, D.E., Ahnine, H., Roumazeilles, L., Santin, M., Ahmed, B., and Lehericy, S. (2019). Offline impact of transcranial focused ultrasound on cortical activation in primates. eLife *8*, e40541.
52. Khalighinejad, N., Bongioanni, A., Verhagen, L., Folloni, D., Attali, D., Aubry, J.-F., Sallet, J., and Rushworth, M.F. (2020). A basal forebrain-cingulate circuit in macaques decides it is time to act. Neuron *105*, 370–384.e8.

CellPress
OPEN ACCESS

STAR★METHODS

KEY RESOURCES TABLE

REAGENT or RESOURCE	SOURCE	IDENTIFIER
Experimental models: Organisms/strains		
Macaca mulatta, 3 males, between x and y years old, between x and y kg, socially housed	MRC, Centre for Macaques	NCBITaxon:9544
Deposited data		
Raw and analysed data	This paper	https://github.com/alimahmoodia/Monkey-Social-Information-Use
Software and algorithms		
MATLAB R2020b	MathWorks	RRID:SCR_001622 https://www.mathworks.com/
FMRIB Software Library v5.0	FMRIB, WIN, Oxford, UK	RRID:SCR_002823 http://www.fmrib.ox.ac.uk/fsl/
Advanced Normalization Tools	Tustison and Avants[45]	N/A
Magnetic Resonance Comparative Anatomy Toolbox	Neuroecology Lab	https://github.com/neuroecology/MrCat
Brainsight	Rogue Research	https://www.rogue-research.com/

RESOURCE AVAILABILITY

Lead contact

Further information and requests for resources should be directed to and will be fulfilled by the lead contact, Ali Mahmoodi (ali.mahmoodi1367@gmail.com).

Materials availability

All data and scripts to replicate the behavioral results are available at https://github.com/alimahmoodia/Monkey-Social-Information-Use. All neuroimaging data are available upon request.

Ethical compliance

All procedures were conducted under licenses from the United Kingdom (UK) Home Office in accordance with the UK Animals (Scientific Procedures) Act 1986 and with the European Union guidelines (EU Directive 2010/63/EU).

Data and code availability

All data and scripts to replicate the behavioral results are available at https://github.com/alimahmoodia/Monkey-Social-Information-Use. All neuroimaging data are available upon request.

METHOD DETAILS

Behavioral training

Three male rhesus macaques (*Macaca mulatta*), aged 9–13 years and weighing 13–17 kg, were involved in the study. The animals, further referred to as T, V, and W, were kept on a 12 h light-dark cycle. MRI-compatible cranial implants (Rogue Research) were surgically implanted to prevent head movements. The macaques were sat in a sphinx position in an MRI-safe chair with their head fixed for the duration of training and testing, using a surgically implanted PEEK headpost (Rogue Research). Responses were recorded using custom-built infrared sensors. Animals had access to water 16–18 h on testing days and with free water access on non-testing days and were deprived of food for 6–8 h before each test or training session.

Stimuli were presented using the Psychophysics Toolbox extension in Matlab.[46]

The macaques were trained to perform binary choices in a computer-based task over the course of 8 months. Training took place in a mock scanner with pre-recorded MRI noise played throughout. First, the animals were trained to associate reward with two objects, one on either side of the screen, before learning the objects' reward probabilities. Next, the animals were taught to follow the direction exhibited by different macaque faces presented on each trial in order to receive reward. The animals were then trained on the three experimental conditions (Experiment 2: fMRI).

Experiment 1: encoding of face direction and identity

In Experiment 1, we conducted a repetition suppression experiment to identify brains areas that encode other animals' face directions. Repetition suppression is the reduction of neural activity due to repeated presentation of a stimulus, or a stimulus feature.[47,48] Here, we looked for brain areas with reduced activity when the direction of two macaque faces presented in close succession was similar compared to when the direction of the second face was dissimilar to the first face. Similarly, we looked for brain areas with reduced activity when the identities of two macaque faces were the same in comparison to when they had different identities. Each monkey completed 10 fMRI sessions of 140 binary choice trials (30 sessions in total, 2 excluded because one animal missed more than 20% of the trials). At the beginning of each trial, a face appeared in the centre of the screen (henceforth, first face). This face could be either *neutral* (gazing forward) or *directed* (gazing left or right). The first face was followed by 500 milliseconds of a blank screen, after which a second, directed face appeared. The animals had 3 seconds from the appearance of the second face to indicate the side the face was gazing at using the infrared sensors. In trials where the monkeys responded prematurely, the trial was terminated and repeated. If the first face was directed, the face direction of the second face was identical to the direction of the first face. However, if the first face was neutral, then the second face was directed either left or right. Additionally, the two faces might have the same identity or different identities. In total, the stimuli comprised 5 different faces, making 5 (identity) by 3 (direction) sets from which faces were selected. We ensured a balanced design by having the same number of trials in each condition of our 2 (same or different direction) by 2 (same or different identity) design. Only the correct trials were included in the final analysis.

Experiment 2: impact of social information on choice

Experiment 2 included 16 fMRI sessions per subject (48 sessions in total), with each session comprising 150 binary choice trials. At the beginning of each trial, a neutral (i.e., gazing forward) macaque face appeared in the centre of a black screen, with an object on either side of the face. After 100ms, the face turned towards one of the objects, and the subjects had 10 seconds to choose one of the two objects by placing either their left or right hand on the corresponding infrared sensor. This decision was then followed by an action-outcome-delay of 3-5 seconds, after which the rewarded object was indicated on the screen and the monkeys either received 2 drops of juice or no juice. Each trial was separated by an inter-trial interval (ITI) of between 3-4 seconds.

Trials were divided into three conditions of 50 trials each. in the Face condition, the face had a reliability of 90%, i.e., the face direction indicated the rewarded object in 90% of the trials. Whereas the object had a reliability of 60%, i.e., one object would result in reward in 60% of the trials while the other would result in reward in 40% of the trials. In this condition, the monkey would receive the most reward if it always followed the face direction. Analogously, in the Object condition, the object had a reliability of 90% while the face had a reliability of 60%. Here, the monkey would receive the most reward if it always chose the most reliable object. In the Mixed condition, both the face and the object had a reliability of 90%, so the monkey could choose whether to follow the face direction or to choose the more reliable of the two objects to obtain the most reward. The proportion of incongruent trials in different conditions were as follows: 39% in the Object condition, 37% in the Face condition, and 17% in the Mixed condition.

Experiment 3: causal manipulation using TUS

The transcranial ultrasound stimulation (TUS) sessions comprised four conditions: three active stimulation sites (the FPC, mSTS, and EVC) and one Sham condition. Each condition was repeated four times, making 16 sessions per animal in total, with the order of sites targeted pseudo-randomised for each animal. There was at least a 24 h gap between each stimulation session to avoid any carryover effect.

Following stimulation, the monkeys were moved to a separate room for their behavioral session. Each behavioral session comprised 100 trials divided into two condition blocks. The two conditions were the *Face* condition and the *Object* condition, differing from the conditions in experiment 2 only by the action-outcome-delay (300ms). Each trial was followed by an inter-trial interval of 3 to 5 seconds. Because animal T and V showed side biases for this experiment, they were forced to press left and right sensors for a drop of juice every seven trials to minimise their side bias.

fMRI data acquisition and processing

MRI data were collected using a 15-coil 3T horizontal bore MRI scanner. Functional data were acquired using a gradient-echo T2* echo planar imaging (EPI) sequence with a 1.25 × 1.25 × 1.25 mm resolution, repetition time (TR) 1.48 s, echo time (TE) 30 ms, flip angle 70°, multiband acceleration factor 2 and GRAPA acceleration factor 2. °. T1-weighted MP-RAGE structural images with a 0.5 x 0.5 x 0.5 mm resolution, TR 2.06 s, TE 4.04 ms, were acquired during a separate session under anaesthesia.

T2* EPI images acquired during the experimental task were reconstructed using a custom offline reconstruction script, using techniques described previously.[49] Preprocessing of structural and functional images involved tools from the FMRIB Software Library (FSL),[50] MATLAB (R2022a, Mathworks), Advanced Normalisation Tools (ANTs; http://stnava.github.io/ANTs),[45] and the Magnetic Resonance Comparative Anatomy Toolbox (MrCat; http://github.com/neuroecology/MrCat).

MP-RAGE images were iteratively pre-processed using a macaque-optimised pipeline using FSL's brain-extraction tool (BET), RF bias-field correction, and linear and non-linear registration (FLIRT and FNIRT) to the *Macaca mulatta* McLaren F99 template implemented in MrCat. The pre-processed structural images of the three animals were then combined to create a group template image. This was accomplished using tools from ANTs as implemented in MrCat, by iteratively registering the initial structural MRI to F99 space, group averaging, and registration of the new group template.

For each session, to correct for distortions of the magnetic field due to motion, each slice of the functional image was linearly and non-linearly registered to a reference based on the EPI volumes from the same timeseries with the least distortion. The aligned and distortion-corrected image for each session was non-linearly registered to the high-resolution structural MRI reference of each subject, which was then registered to the group template using ANTs. EPI images were spatially smoothed (3 mm FWHM) and temporally high pass filtered (cut-off 100 s).

TUS protocol

Transcranial ultrasound stimulation (TUS) was performed using a four-element annular array transducer (NeuroFUS (CTX-250, 64mm active diameter, Brainbox Ltd, Cardiff, UK) combined with a programmable amplifier (Transducer Power Output System, TPO-105, Brainbox Ltd, Cardiff, UK). The transducer was paired with a transparent coupling cone filled with degassed water and sealed with a latex membrane. The resonance frequency of the ultrasonic wave was set to 250kHz. The stimulation comprised 30ms bursts of ultrasound), for a total stimulation duration of 40s. The stimulation protocol followed previously established protocols in macaques,[51,52] delivered through a Transducer Power Output (TPO) system (Sonic Concepts). The spatial-peak pulse-average intensity (I_{SPPA}) in water (free field) was 60.0 W/cm^2. This intensity is attenuated when passing through the skull.

At the beginning of each stimulation session the animal's skull was shaved and a conductive gel (SignaGel Electrode; Parker Laboratories Inc.) was applied to the skin. The water-filled coupling cone and the gel were used to ensure ultrasonic coupling between the transducer and the animal's head. Next, the ultrasound transducer / coupling cone montage was placed on the skull and a Brainsight Neuronavigation System (Rogue Research, Montreal, CA) was used to position the montage so that the focal spot was centered on the targeted brain region. All targets were sonicated bilaterally for 80 s in total, with 40 s of stimulation applied to a target in each hemisphere (except for FPC which was only stimulated for 40s as the target was on the midline). Sonication of the target in one hemisphere was immediately followed by sonication of a homologous target in the contralateral hemisphere. Hemispheres were sonicated in a pseudo-random order. After stimulation, monkeys were immediately moved to a testing room for behavioral data collection.

There were four stimulation conditions: the frontal polar cortex (FPC) [0, 27, 9] the medial superior temporal sulcus (mSTS) [25.2, -15.6, -0.5] and [-22.0, -14.6, -4.6], the extrastriate visual cortex (EVC) [23, -35, -4] and [-23, -35, -4], and Sham. Note that the precise EVC coordinate that we reported in our fMRI results was not accessible to the TUS coil. We therefore used the coordinate mentioned above which was associated with qualitatively identical activity patterns to those seen at the area of peak change reported in the fMRI Results sections. The three active stimulation sites were projected onto individual T_1-weighted MRI brain scans using the Brainsight Neuronavigation System to inform accurate online positioning of the transducer over each target region during stimulation. The Sham sessions, included as a non-stimulation passive control, were identical to the active stimulation sessions, except that the sonification was not applied. During the sham sessions, the ultrasound transducer was randomly placed over one of the target sites.

QUANTIFICATION AND STATISTICAL ANALYSIS

Behavioral data analysis

Linear mixed effect models

In all linear regression models (see below for details), we assessed the statistical significance of model parameters by F-statistics and Satterthwaite's approximation for degrees of freedom.

LMM1

To investigate the effect of the social and object information on choice in experiment 2, we conducted a linear mixed effect model as follow:

$$choice_t = \beta_{1s} + \beta_{2s} \times face\ direction_t + \beta_{3s} \times object\ direction_t + \beta_{4s} face\ direction_t \times object\ direction_t \quad \text{(Equation 1)}$$

Where $choice_t$ indicates animals' choice on trial t and was set to 1 if left was selected and 2 otherwise. $face\ direction_t$ and $object\ direction_t$ indicate the direction of the face and the side of the object with the higher probability of reward in trial t (1 for left and 2 for right), respectively. The intercept (β_{1s})and all slopes (β_{ks}) were allowed to vary across sessions (denoted by s) by including random effects of the form $\beta_{ks} = \beta_{k0} + b_{ks}$ where $b_{ks} \sim N(0, \sigma_k^2)$.

LMM2

To investigate the effect of incongruency on accuracy in experiment 2, we conducted a linear mixed effect model as follow:

$$accuracy_t = \beta_{1s} + \beta_{2s} \times congruency_t \quad \text{(Equation 2)}$$

Where $accuracy_t$ indicates animals' accuracy in trial t (0 and 1 for wrong and correct responses, respectively), and $congruency_t$ indicates congruency in trial t and was set to 0 for a congruent trial and to 1 for an incongruent trial. The intercept (β_{1s})and β_{2s} were allowed to vary across sessions (denoted by s) by including random effects of the form $\beta_{ks} = \beta_{k0} + b_{ks}$ where $b_{ks} \sim N(0, \sigma_k^2)$.

LMM3

For analysis of experiment 3 (TUS experiment), for each of the two trial types (congruent and incongruent), we ran a linear mixed-effects model to investigate the effect of stimulation on accuracy. To this end we compared each active stimulation site (FPC, mSTS, EVC) versus sham separately for congruent and incongruent trials and for each condition (Face and Object) as follows:

$$accuracy_t = \beta_{1s} + \beta_{2s} \times ROI_t \quad \text{(Equation 3)}$$

Where $accuracy_t$ indicates animals' accuracy in trial t (0 and 1 for wrong and correct responses, respectively), and ROI_tindicates the ROI/condition of stimulation in trial t and was set to 0 for all trials in the Sham sessions and to 1 for all trials in the active stimulation sessions. The intercept (β_{1s})and β_{2s} were allowed to vary across sessions (denoted by s) by including random effects of the form $\beta_{ks} = \beta_{k0} + b_{ks}$ where $b_{ks} \sim N(0, \sigma_k^2)$.

Where the Hessian Matrix of the model was not positive definite, potentially due to overparameterization of the model, we changed all the mixed effect variables in Equation 3 to fixed effect to ascertain a positive definite Hessian matrix which is indispensable for reliable beta estimation.

We used the same model (Equation 3) to compare active sessions against each other. For this purpose, we included two active stimulation sessions (e.g., FPC and EVC) in the model instead of one active stimulation session and one Sham session.

LMM4

For each of the two trial types (congruent and incongruent), we ran a linear mixed-effects model to investigate the effect of stimulation on the contribution of social information on choice. To this end we compared each active stimulation site (FPC, mSTS, EVC) versus sham separately for congruent and incongruent trials and for each condition (Face and Object) as follows:

$$choice_t = \beta_{1s} + \beta_{2s} \times face\ direction_t + \beta_{3s} \times ROI_t + \beta_{4s} \times face\ direction_t \times ROI_t \quad \text{(Equation 4)}$$

Where $choice_t$ indicates animals' choice on trial t and was set to 1 if left was selected and 2 otherwise. $face\ direction_t$ indicates the direction of the face in trial t (1 for left and 2 otherwise).ROI_t indicates ROI/condition of stimulation in trial t and was set to 0 for all trials in the Sham sessions and to 1 for all trials in the active stimulation sessions. The intercept (β_{1s})and all slopes (β_{ks}) were allowed to vary across sessions (denoted by s) by including random effects of the form $\beta_{ks} = \beta_{k0} + b_{ks}$ where $b_{ks} \sim N(0, \sigma_k^2)$.

To investigate the effect of object information on choice, we replaced $face\ direction_t$with $object\ direction_t$ in Equation 4, where $object\ direction_t$indicates the direction of the object with the higher probability of reward in trial t (1 for left and 2 for right).

In all linear mixed-effect models, the model parameters were estimated using Maximum Likelihood method of fitting. After running each model, we confirmed the normality assumption of the model by running a Kolmogorov-Smirnov test.

We initially included all variables as random effects, but if the Hessian Matrix of the model was not positive definite, potentially due to overparameterization of the model, we changed the mixed effect variables to fixed effect to ascertain a positive definite Hessian matrix which is indispensable for reliable estimation.

In all models that we conducted for the Face and Object conditions we always compared the full model (which included both face and object direction and their interaction) with a simpler model which only contained the more reliable information on that block using likelihood ratio test. In all cases, the full model provided the best model fit.

fMRI data analysis

fMRI data were analysed using FSL. The whole-brain functional data was analysed in FEAT (FMRI Expert Analysis Tool) Version 6.00, part of FSL, using a univariate general linear model (GLM) approach. At the first level, FEAT computed contrasts of parameter estimates, and variance estimates for each contrast. These estimates were then transformed into standard space using ANTs. Higher-level analyses utilised FLAME 1+2 (FMRIB's Local Analysis of Mixed Effects, stage 1 and 2) and treated sessions as random effects.

In experiment 1, two sessions were excluded from the fMRI analyses because monkey V did not finish the sessions. Therefore, experiment 1 comprised 28 total sessions. In experiment 2, two sessions were excluded from the analyses (one session from T and one session from W) due to technical issues. Thus, experiment 2 comprised 46 sessions.

GLMs 1 & 2 (whole brain analysis for experiment 1)

For the whole-brain analysis of experiment 1, we conducted the following univariate GLMs. GLM1 was conducted to test for brain areas that encoded face direction:

$$\begin{aligned} BOLD = {} & \beta_1 face + \beta_2 dsimFace + \beta_3 simFace + \beta_4 RT + \beta_5 leftResp + \beta_6 rightResp + \beta_7 outcome \\ & + \beta_8 binOutcome + \beta_9 leftUnconv + \beta_{10} rightUnconv + \beta_{11} juice + \beta_{12} fchInvalid + \beta_{13} schInvalid \end{aligned} \quad \text{(Equation 5)}$$

Regressors 1-8 and 12-13 were HRF-convolved with a gamma function (mean lag 3s, standard deviation 1.5s). Here, *BOLD* is a t x 1 (t time samples) column vector containing the time series data for a given voxel. *face* represents the onset of the first face (constant regressor coded as 1). In the face direction analysis, *dsimFace* and *simFace* represent the decision onset when the second face direction was dissimilar or similar to the first face, respectively. *RT* represents the reaction time in all trials, time-locked to decision onset and z-scored. *leftResp* and *rightResp* are regressors aligned to the decision onset in trials in which a left- or right-hand

movement was recorded, respectively. *outcome* is the main effect of outcome (constant regressor encoded as 1) time-locked to the onset of feedback (i.e., juice). *binOutcome* ∈ (1,0) represents the effects of the outcome and was assigned 1 if the macaque received juice reward, and 0 if they answered incorrectly. It was time-locked to the onset of feedback. *leftUnconv*, *rightUnconv*, and *juice*, are unconvolved regressors that model signal distortions in the magnetic field due to movement, and so are not convolved with HRF. *fchInvalid* and *schInvalid* model the onset of the first and second face on all invalid trials. A trial was labelled invalid if the animal failed to respond at all or the response was slower than 1.5 seconds for animals V and W or 2.5 seconds for animal T (because this animal had a slower reaction time). In addition, task-unrelated confound regressors were included in the model to further reduce variance and noise in the BOLD signal. These regressors comprised 13 parametric PCA components describing the warping from each volume to the mean reference image when correcting for distortion in the magnetic field due to motion.

GLM2 was conducted to test for brain areas that encoded face identity. This GLM differed from GLM1 in only two regressors: regressors 2 and 3 modelled trials in which the second face was similar or dissimilar to the first face in terms of identity (instead of face direction).

The duration of all convolved regressors was a boxcar of 500ms. The duration of all unconvolved regressors was one TR.

GLM 3 (whole brain analysis for experiment 2)

For the whole brain analysis of experiment 2, we conducted the following GLM:

$$BOLD = \beta_1 object + \beta_2 face + \beta_3 congruent + \beta_4 noResp + \beta_5 RTvalid + \beta_6 RTinvalid + \beta_7 leftResp + \beta_8 rightResp + \beta_9 binOutcomeObject + \beta_{10} binOutcomeFace + \beta_{11} binOutcomeCong + \beta_{12} leftUnconv + \beta_{13} rightUnconv + \beta_{14} juice \quad \text{(Equation 6)}$$

Regressors 1-11 were HRF-convolved with a gamma function (mean lag 3 s, standard deviation 1.5s). In Equation 6, *BOLD* is a t x 1 (t time samples) column vector containing the time series data for a given voxel. *object* and *face* represent the main effects at the time of decision in incongruent trials where the object or face was followed, respectively, while *congruent* represents the main effects at the time of decision in congruent trials. *noResp* represents trials where no response was received. *RTvalid* and *RTinvalid* represent the reaction times in valid and invalid trials, respectively, where invalid trials are trials in which the animals took > 10 seconds to respond. These regressors were time-locked to decision onset and z-scored. *leftResp* and *rightResp* are regressors aligned to the decision onset of trials in which a left- or right-hand movement was recorded. *binOutcomeObject*, *binOutcomeFace*, and *binOutcomeCong* are binary {1,0} regressors representing main effects at the time of outcome in incongruent object and face trials, and congruent trials, respectively. The regressor was coded 1 if the animal received reward and 0 if they did not. The final three regressors (*leftUnconv*, *rightUnconv*, *juice*) were unconvolved. These conditions caused changes in the magnetic field due to movement, and thus the regressors model the instant signal distortions rather than neural activity. As before, 13 task-unrelated confound regressors were also added to the model. For all the convolved regressors, we used a boxcar of 1 second in length. The duration of outcome related regressors was 2 seconds. For the duration of non-convolved regressors we used a boxcar of one TR in length.

Activity time course analysis

For each scan session, we regressed out variation due to head motion, and up-sampled the BOLD time course to a resolution of 10 samples per TR. For each trial, we extracted activity time-locked to the onset of each event of interest up to 7 seconds after the onset of the event. We used linear regression to predict the ROI activity time courses. More specifically, we applied a linear regression to each time point and then, by concatenating beta-weights across time points and averaging across voxels, created a beta-weight time course for each predictor of a regression model. We performed this step separately for each session and pooled beta-weight time courses across sessions and subjects for visualisation. We then computed the average across all sessions as the observed beta weights. The significance of each time points from zero was determined using a permutation test. For a given time point, separately for each session, we shuffled the trial labels (preserving the number of trials) and computed the regression beta for each time point and session. We repeated this procedure 1,000 times to establish the null distribution. After each repetition, we computed the average across all sessions. We subsequently compared the shuffled beta weights to the observed beta weights to report the *p*-value. We computed the *p*-value from the onset of each event until the monkey hemodynamic response function reaches its peak (i.e., 3 seconds after the onset of each event). We then calculated the FDR-corrected *p*-values and reported the FDR-corrected *p*-values in the main text.

We used Equation 7 below for performing time course analyses pertaining to repetition suppression analysis, Figures 1B–1D:

$$BOLD_t = \beta_0 + \beta_1 \times trial\ type_t + \beta_2 \times RT_t \quad \text{(Equation 7)}$$

Where $BOLD_t$ indicates the BOLD response on trial t. $Trial\ type_t$ indicates whether the direction of the two faces were similar or dissimilar on trial t. It was set to 1 if the direction of the two faces in trial t were dissimilar and 0 otherwise.

We used Equation 8 below for performing time course analyses pertaining to Figures 3D–3F:

$$BOLD_t = \beta_0 + \beta_1 \times trial\ type_t + \beta_2 \times RT_t \quad \text{(Equation 8)}$$

Where $BOLD_t$ indicates the BOLD response on trial t. $Trial\ type_t$ indicates whether the trial was incongruent or congruent on trial t. It was set to 1 if the trial was incongruent and 0 otherwise.

We used Equation 9 below for PPI pertaining to Figures 3G and 3H:

$$dmFPC\ BOLD_t = \beta_0 + \beta_1 \times incongruency_t + \beta_2 \times physio_t + \beta_3 \times incongruency_t \times physio_t \quad \text{(Equation 9)}$$

Where $dmFPC\ BOLD_t$ is dmFPC BOLD response on trial t. $Incongruency_t$ indicates whether a trial was incongruent and was set to 1 if so and 0 otherwise. $Physio_t$ indicates BOLD response of the physiological variable on trial and was extracted from mSTS or EVC for creating Figures 3G and 3H, respectively.

Creating Region of Interests (ROIs) for the time course analysis

The coordinate for creating mSTS, lateral mSTS and AM were taken from a previous study[23] as follows (all coordinates in F99 standard space [x, y, z]): mSTS (25.2, -15.6, -0.5; -22.0, -14.6, -4.6), lateral mSTS (30.7; -16.1, 4.5; -27.6, -17.2, 2.5), AM (22.1, -1.5, -16.3; -20.5, -07, -14.7). The ROIs for FPC was created by defining a spherical mask (r=2mm) centred approximately around the centre of the cluster (F99 standard space [x, y, z] 0, 27, 12) which was significantly positive in incongruent Face versus Object condition obtained in the whole brain analysis using GLM3. The ROIs for EVC were created by defining a spherical mask (r=2mm) centred approximately around the centre of the cluster (F99 standard space [x, y, z] 23, -35, -4) which was significantly negative in the incongruent Face versus Object condition obtained in the whole brain analysis employing GLM3.

Neuron, Volume *112*

Supplemental information

A frontopolar-temporal circuit determines the impact of social information in macaque decision making

Ali Mahmoodi, Caroline Harbison, Alessandro Bongioanni, Andrew Emberton, Lea Roumazeilles, Jerome Sallet, Nima Khalighinejad, and Matthew F.S. Rushworth

Supplementary Information

A frontopolar-temporal circuit determines the impact of social information in macaque decision-making

Primary Supplemental PDF:

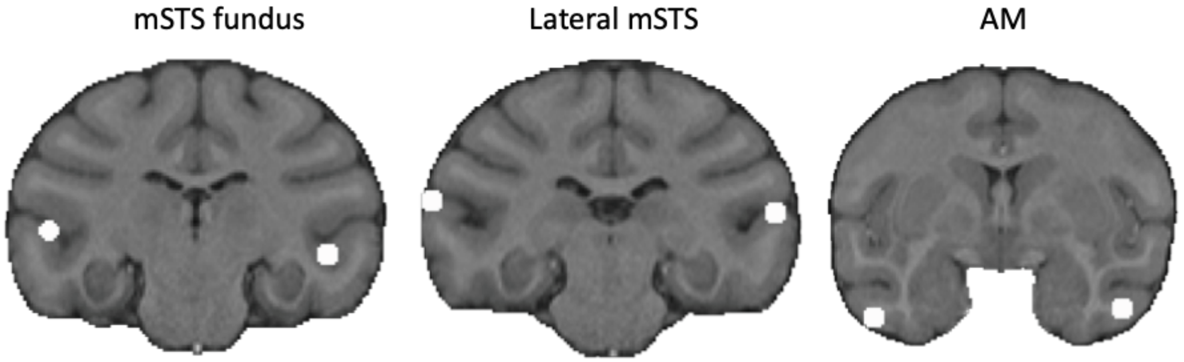

Figure S1: ***ROIs which were considered for experiment 1, related to Figure 1.*** **a** Coronal view of the three ROIs that we investigated for experiment 1. F99 standard space x, y, z coordinates for fundus mSTS: [25.2, -15.6, -0.5]; [-22.0, -14.6, -4.6], for lateral mSTS [30.7; -16.1, 4.5]; [-27.6, -17.2, 2.5] and for AM [22.1, -1.5, -16.3]; [-20.5, -.7, -14.7]. To analyse the data *(***Repetition suppression analyses**), We optimised our design to exploit repetition suppression (a reduction in neural activity due to repeated presentation of a stimulus feature), to find brain areas encoding face direction by looking for areas in which activity was reduced when the second face direction was the same as, as opposed to different to, that of the first face. For this purpose, we ran a time course analysis at the time of the second face in which we regressed blood oxygen level dependent (BOLD) response as dependent variable onto face "direction similarity" and reaction time (as a confound variable) as independent variables. For each trial, direction similarity was set to 0 if the first and the second faces were gazing in the same direction, and to 1 otherwise. Therefore, if an area responds positively to this regressor, it means that it is exhibiting face direction-dependent repetition suppression. As explained in the methods of experiment 1, in addition to face direction, the two faces might have the same identity or different identities. In total, the stimuli comprised 5 different faces, making 5 (identity) by 3 (direction) sets from which faces were selected. We ran whole-brain GLMs to identify brain regions that showed significant repetition-suppression to face direction or face identity (see GLM1 and GLM2 in Methods). No area was significantly modulated by face direction at the whole brain level. One area, in posterior STS, was significantly modulated by face identity at the whole brain level, MNI coordinate [-22.6 -21.1 7.04], peak z-value 3.5, cluster size: 2043 (all coordinates correspond to the F99 atlas[1]).

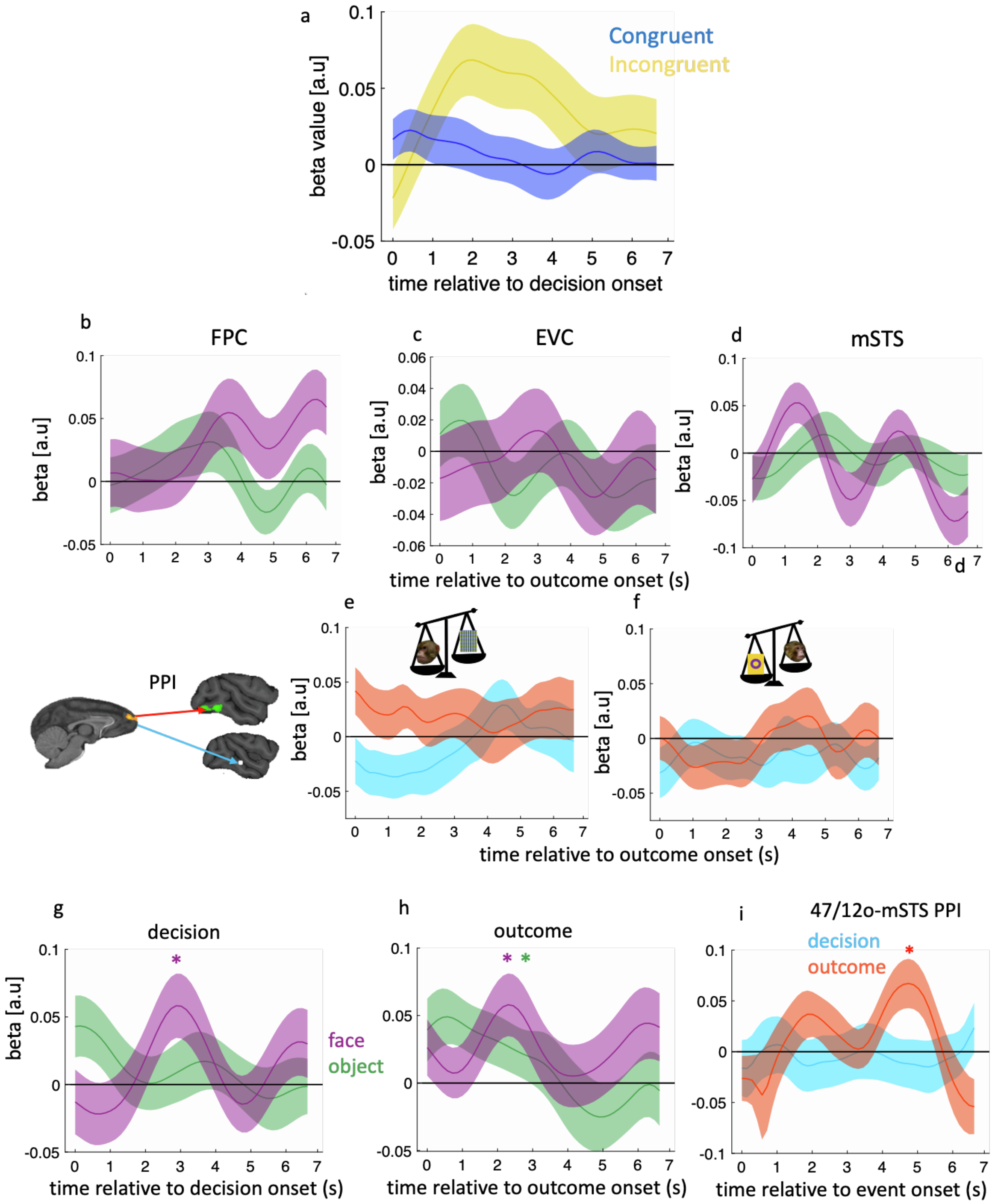

Figure S2: **Specificity of dmFPC activity incongruent trials at decision time, related to Figure 3. a** In experiment 2, We investigated the difference between the Face and Object conditions separately for the congruent and incongruent trials. To this aim, we ran a regression model in which we regressed dmFPC BOLD (as the dependent variable) against condition (coded as 1 for Face and 0 for Object, respectively) and reaction time. We repeated this procedure separately for the congruent (blue curve) and incongruent (yellow curve) trials. Consistent with our whole brain GLM in the main text (GLM3), we found that in dmFPC activity was significantly higher in the Face versus Object condition, but only in incongruent trials (W=765, p=.01), but not in congruent trials (W=639, p=.28). ***b-f*** Analogous to Figure 3 in the main text but at outcome time. dmFPC, EVC, and mSTS activity at outcome time and their functional

connectivity with other areas are different from decision time. None of the areas respond to incongruency at outcome time (b, c, d). In addition, there is no functional connectivity between dmFPC and EVC or dmFPC and mSTS neither in Face (e) nor in Object (f) condition at the time of outcome. ***g-i*** To investigate the specificity of the dmFPC activity and functional connectivity pattern that we observed, we repeated our analysis in another area of the frontal cortex, area 47/12o. Given the importance of this area in learning and credit assignment[2–7], we expected 47/12o's role, unlike dmFPC's, to be more specific to outcome time as opposed to decision time. We therefore expected its activity to differ from what we had observed in dmFPC. We extracted 47/12o's time course at both decision and outcome times and regressed it against incongruency. At decision time, 47/12o activity was significantly above zero in the incongruent Face condition (panel g, W=720, p=.04, HBC), but not Object condition (panel g, W=698, p=.08, HBC) and its activity in the incongruent trials was significantly higher in the Face than the Object condition (W=771, p=.01). Comparing its activity with dmFPC, we found that 47/12o's activity arose approximately 2s later, but its peak level of activity was not significantly different from dmFPC at decision time in the Face condition (W=521, p=.83), while they were significantly different in the object condition (W=746, p=.02). At the time of outcome, the 47/12o response to incongruency was significantly positive in both Face and Object conditions (panel h, Face W=779, p=.009, Object W=716, p=.05). However, its response to incongruency was higher in the Face compared to the Object condition (W=780, p=.008). In addition, the activity of 47/12o at outcome time in the Face condition (panel h) was significantly higher than dmFPC at outcome time (W=305, p=.01). To understand how it played this role, just as we had done for dmFPC, we examined 47/12o's functional connectivity (as seed region) with mSTS (as physiological variable) with incongruency as the psychological variable. Given the higher response of 47/12o to the Face condition and mSTS involvement in encoding face direction, we restricted analysis to the Face condition. Unlike for dmFPC-mSTS, there was no change in connectivity between 47/12o and mSTS at decision time (panel i, blue curve, W=320, p=.99, HBC) but their connectivity significantly increased at outcome time (panel i, red curve, W=749, p=.04, HBC). Finally, connectivity between dmFPC-mSTS was significantly higher than 47/12o-mSTS at decision time (W=273, p=.003), while there was a trend toward the opposite direction at the outcome time (W=399, p=.12).
To perform the ***Functional connectivity analyses,*** we performed the following procedures. Functional connectivity between two brain regions can be assessed using psychophysiological interaction (PPI). In a PPI analysis, activity of a brain region (seed region) is predicted using the interaction between activity of another brain region (physiological variable) and a psychological variable, while controlling for both main effects (physiological and psychological variables). An issue when doing functional connectivity analysis is to what degree the areas involved in the analysis are anatomically connected to each other. Most anatomical connectivity studies regarding mSTS have emphasized connections with the dorsal bank of the STS rather than the fundus and adjacent ventral bank that is the focus of the current study. However, because at each rostrocaudal level of the STS there are strong connections between each STS area and the laterally and medially adjacent STS areas[8], there is a two stage corticocortical route between dmFPC and mSTS. Similarly, there are no direct connections between dmFPC and the extrastriate areas, such as areas V4/TEO at the centre of the EVC region. EVC was linked to the guidance of decision making by visual object identity as opposed to faces. There are, however, other two-stage corticocortical routes known to influence activity in EVC that might run via ventrolateral prefrontal cortex[9–12]. Intriguingly, however, there are also subcortical routes by which dmFPC might modulate mSTS and EVC activity, for

example via thalamic nuclei such as the pulvinar, where face-responsive neurons have also been reported[13], which is connected with dmFPC, mSTS and EVC[14,15], and which is known to modulate visual activity[14,16].

			Peak MNI coordinates		
**Activation**	**Cluster size**	**Z**	**x**	**y**	**z**
		**Face minus object at decision time**			
Positive	2155	4.52	8.55	27.7	14.1
Negative	6206	4.05	2.01	-43.3	2.01
	3802	4.69	14.6	-41.7	-9.05
		**Face minus object at outcome time**			
Positive	21814	8.08	14.1	16.1	16.1
	2175	4.32	27.7	1.51	-6.04
Negative	15535	5.96	-27.7	0.5	12.6
	5261	4.02	-4.02	25.7	2.52
	4449	3.93	14.1	-1.51	0
	2880	4.39	-3.52	-12.1	-1.51
	1691	3.74	26.2	-6.54	10.1
		**Incongruent minus congruent trials at decision time**			
Positive	2236	3.49	-1.51	-25.7	4.02
		**Incongruent minus congruent trials at outcome time**			
Positive	11541	5.97	23.1	-3.52	14.6
	9086	5.47	-23.6	-2.01	9.05
Negative	1744	3.71	-1.01	16.1	1.51

Table S1: Whole brain results obtained from GLM3, related to Figure 3.

ROI	Condition	Beta±CI	F statistic	P-value
dmFPC vs Sham	Face	-.004±.06	.06	.80
STS vs Sham	Face	-.01±.07	.51	.47
EVC vs Sham	Face	.01±.06	.70	.40
dmFPC vs Sham	Object	-.01±.03	2.54	.11
STS vs Sham	Object	-.003±.02	.32	.57
EVC vs Sham	Object	-.003±.02	.34	.56

Table S2: TUS effect on congruent trials, related to Figure 4. Effect of TUS on congruent trials in the Face and Object conditions obtained by running LMM3. All p-values uncorrected.

ROI	Condition	Coefficient	Beta±CI	F statistic	P value
mSTS	Object	Object	.49±.07	690.08	<.001
		Face	.01±.05	.99	.32
	Face	Object	.01±.12	.15	.70
		Face	.89±.07	2411.70	<.001
dmFPC	Object	Object	.49±.12	250.21	<.001
		Face	.08±.08	17.11	<.001
	Face	Object	.04±.02	.52	.47
		Face	.80±.12	645.13	<.001
EVC	Object	Object	.47±.08	517.31	<.001
		Face	.04±.05	9.42	.002
	Face	Object	.02±.09	.95	.33
		Face	.94±.06	4431.00	<.001
Sham	Object	Object	.47±.05	1384.80	<.001
		Face	.01±.03	2.39	.12
	Face	Object	-.01±.10	.19	.67
		Face	.94±.06	3789.70	<.001

Table S3: Effect of face and object information on choice for Face and Object conditions, following each TUS condition, related to Figure 4. Our TUS results reported in the main text indicates that the effect of more reliable information in each condition was reduced following dmFPC TUS. To directly show this effect, we conducted another linear mixed effect model (see LMM4 in Methods) on incongruent trials separately for Face and Object conditions and compared each active stimulation condition versus Sham. In this model, the animals' choice was introduced as the dependent variables were (1) the direction of the reliable information in each condition (0 and 1 for left and right side), (2) ROI (0 for sham and 1 for any other active stimulation condition), and the interaction between these two regressors. It should be noted that this analysis is a confirmatory analysis and is not orthogonal to our accuracy analyses which we presented above and does not provide any information above and beyond suggested by the previous analysis but clarifies its interpretation. Consistent with our accuracy analysis, we found that in dmFPC TUS the effect of face was significantly lower than Sham in the incongruent trials of the Face condition, ($\beta\pm95\%$ CI=-.15±.09, $F(1,546)=14.3$ $p<.001$,HBC) and also than EVC ($\beta\pm95\%$ CI=-.11±.09, $F(1,547)=6.44$ $p=.01$,HBC). The effect of the face was also lower after mSTS TUS than Sham ($\beta\pm95\%$ CI=-.08±.06, $F(1,550)=7.38$ p<. 01). Similarly, in the incongruent trials of the Object condition, the face had a larger effect on choice after dmFPC TUS than Sham ($\beta\pm95\%$ CI=.04±.03, $F(1,584)=6.18$ $p=.01$,HBC) and also EVC compared to Sham ($\beta\pm95\%$ CI=-.03±.03, $F(1,579)=4.72$ $p=.03$, HBC). Finally, we repeated our LMM1 (the linear mixed effect model that we used to analyse our behavioural data of experiment 2) separately for each condition (Face or Object) and each TUS condition (Sham, dmFPC, mSTS, Sham). Consistent with the result of experiment 2, we found that in all conditions there was a significant impact of the more reliable information on choice (See Table).

Condition	Face	Object
Following correct trials	88±6(%)	94±5(%)
Following error trials	83 ± 19(%)	94±12(%)

Table S4: Investigating the effect of learning in experiment 2, related to Figure 2.

As we mentioned in our task description, the animals were tested once they reached near optimal performance. The animals' performance in the task (shown in Figure 2) supports this claim. Therefore, the computational problem that the animals encountered during the task was to weigh each source of information according to its reliability, but not learning the reliabilities. The reliability of each source of information was pre-learned during training. The weights could have been stored in terms of Q-values if we assume a reinforcement learning algorithm was recruited by the animals during training. It could be argued that the animals updated the weights during the task. However, if the animals updated the pre-learned weights during the task or were following a win-stay-lose-shift (WSLS) strategy, we would expect an effect of previous outcome on the current choice (effect of outcome on trials t-1 on the choice on trial t). In addition, if the animals were learning the task, we would expect their choice on the very first trial of each condition to be random. We tested both predictions. First, on the very first trial of the Face condition, the animals followed the face in 42 out of 46 sessions. The animals followed the better object in 42 out of 46 sessions in the first trial of the Object condition as well. These results indicate that the animals had learned the value/reliability of each source of information almost perfectly before doing the task, consistent with our training procedure and our claim in the manuscript. It should be noted that we used different pairs of objects and faces for reliable and unreliable objects and conditions, respectively.

We then went on to test whether there was any effect of previous outcome on choice or any evidence for WSLS strategy in the animals' behaviour. We first computed the proportion of trials on which animals chose the more reliable source of information on each condition (face direction in the Face condition and object direction in the Object condition) separately for trials on which the reliable source of information was correct or wrong on the previous trial. We argued that if the animals updated their value estimate after observing an outcome, we would expect the animals to choose the alternative information more often after wrong compared to correct trials. We found that in the Face condition in 83 ± 19(%) (mean ± standard deviation) of the trials the animals chose the face direction again after it had led to the wrong choice in the previous trial. Obviously, this is significantly above chance level ($w=893$, $p<.0001$). In the same condition, the animals followed the face direction after it led to the correct choice in the previous trial in 88±6(%) (m±std) of the trials. Comparing choosing face direction following correct and wrong trials showed no significant difference between the two trial types ($w=432$, $p=.32$).

We observed the same pattern in the Object condition: In 94±12(%) (m±std) of the trials the animals chose the same object that led to the wrong choice in the previous trial (test against chance level, $w=990$, $p<.0001$). The animals followed the same object after it led to the correct choice in the previous trial in 94±5(%) (m±std) of the trials. Again, with no difference in choosing the same object between the two trial types ($W=287$, $p=.76$).

These results indicate that there was no effect of previous outcome on current choice, consistent with the suggestion that while object and face values may initially have been

malleable when they were first encountered and during learning, this was no longer the case by the end of our training procedure. By this stage animals had learned the value/reliability of the face cues and the object cues which remained constant. Finally, we combined both conditions and conducted a regression model in which we predicted the animals' choices following the more reliable information on the current trial as a function of its accuracy on the previous trial. We conducted this model on the first 10 trials of each condition, where any potential learning is more likely to happen. Consistent with the analysis that we reported above, there was no effect of previous outcome on current choice (beta±95%CI= 0±.22, p=.93).

Supplementary references:

1. Van Essen, D.C., and Dierker, D.L. (2007). Surface-based and probabilistic atlases of primate cerebral cortex. Neuron *56*, 209–225.

2. Chau, B.K., Sallet, J., Papageorgiou, G.K., Noonan, M.P., Bell, A.H., Walton, M.E., and Rushworth, M.F. (2015). Contrasting roles for orbitofrontal cortex and amygdala in credit assignment and learning in macaques. Neuron *87*, 1106–1118.

3. Folloni, D., Fouragnan, E., Wittmann, M.K., Roumazeilles, L., Tankelevitch, L., Verhagen, L., Attali, D., Aubry, J.-F., Sallet, J., and Rushworth, M.F. (2021). Ultrasound modulation of macaque prefrontal cortex selectively alters credit assignment–related activity and behavior. Sci. Adv. *7*, eabg7700.

4. Rudebeck, P.H., Saunders, R.C., Lundgren, D.A., and Murray, E.A. (2017). Specialized representations of value in the orbital and ventrolateral prefrontal cortex: desirability versus availability of outcomes. Neuron *95*, 1208–1220.

5. Murray, E.A., and Rudebeck, P.H. (2018). Specializations for reward-guided decision-making in the primate ventral prefrontal cortex. Nat. Rev. Neurosci. *19*, 404–417.

6. Tsujimoto, S., Genovesio, A., and Wise, S.P. (2009). Monkey orbitofrontal cortex encodes response choices near feedback time. J. Neurosci. *29*, 2569–2574.

7. Miller, K.J., Botvinick, M.M., and Brody, C.D. (2022). Value representations in the rodent orbitofrontal cortex drive learning, not choice. Elife *11*, e64575.

8. Seltzer, B., and Pandya, D.N. (1989). Intrinsic connections and architectonics of the superior temporal sulcus in the rhesus monkey. J. Comp. Neurol. *290*, 451–471.

9. Yeterian, E.H., Pandya, D.N., Tomaiuolo, F., and Petrides, M. (2012). The cortical connectivity of the prefrontal cortex in the monkey brain. Cortex *48*, 58–81.

10. Gregoriou, G.G., Rossi, A.F., Ungerleider, L.G., and Desimone, R. (2014). Lesions of prefrontal cortex reduce attentional modulation of neuronal responses and synchrony in V4. Nat. Neurosci. *17*, 1003–1011.

11. Bichot, N.P., Xu, R., Ghadooshahy, A., Williams, M.L., and Desimone, R. (2019). The role of prefrontal cortex in the control of feature attention in area V4. Nat. Commun. *10*, 5727.

12. Xu, R., Bichot, N.P., Takahashi, A., and Desimone, R. (2022). The cortical connectome of primate lateral prefrontal cortex. Neuron *110*, 312–327.

13. Nguyen, M.N., Hori, E., Matsumoto, J., Tran, A.H., Ono, T., and Nishijo, H. (2013). Neuronal responses to face-like stimuli in the monkey pulvinar. Eur. J. Neurosci. *37*, 35–51.

14. Saalmann, Y.B., Pinsk, M.A., Wang, L., Li, X., and Kastner, S. (2012). The pulvinar regulates information transmission between cortical areas based on attention demands. science *337*, 753–756.

15. Romanski, L.M., Giguere, M., Bates, J.F., and Goldman-Rakic, P.S. (1997). Topographic organization of medial pulvinar connections with the prefrontal cortex in the rhesus monkey. J. Comp. Neurol. *379*, 313–332.

16. Boshra, R., and Kastner, S. (2022). Attention control in the primate brain. Curr. Opin. Neurobiol. *76*, 102605.
